# Eye movements of children with and without developmental dyslexia in an alphabetic script during alphabetic and logographic tasks

**DOI:** 10.1038/s41598-024-78894-2

**Published:** 2024-11-20

**Authors:** Susanne Trauzettel-Klosinski, Theda Faisst, Vera Schick, Giulia Righetti, Christoph Braun, Angelika Cordey-Henke, Ching-Chu Sun, Stephan Kuester-Gruber

**Affiliations:** 1 Vision Rehabilitation Research Unit, Center for Ophthalmology, University of Tuebingen, Tuebingen, Germany; 2 Erich-Paulun- Institute, China Center Tuebingen, University of Tuebingen, Tuebingen, Germany; 3 Center for Ophthalmology, University Eye Hospital, University of Tuebingen, Tuebingen, Germany; 4 MEG-Center, University of Tuebingen, Tuebingen, Germany; 5 Hertie Institute for Clinical Brain Research, University of Tuebingen, Tuebingen, Germany; 6 DiPSCO, Department of Psychology and Cognitive Science, University of Trento, Rovereto, Italy; 7 Center for Mind/Brain Sciences, University of Trento, Rovereto, Italy; 8 Department of Quantitative Linguistics, University of Tuebingen, Tuebingen, Germany; 9 Department of Cognitive Science and Artificial Intelligence, Tilburg University, Tilburg, The Netherlands

**Keywords:** Developmental dyslexia, Alphabetic writing system, Logographic writing system, Eye Movements, Visuo-spatial pathway, Phonological encoding, Dyslexia, Paediatric research

## Abstract

Eye movements (EM) during naming alphabetic versus logographic stimuli in children with and without developmental dyslexia (DD) were examined for each stimulus separately to identify conspicuous characteristics that influence naming performance. 40 children (group DD = 18; control group C = 22) were taught Chinese characters. EM were recorded during naming alphabetic words, pictures and Chinese characters. Main variables were articulation latencies, numbers and durations of fixations, secondary variables were fixation locations and error rates. Group DD showed significantly longer latencies and more fixations while reading words, but only insignificantly more fixations while naming pictures and Chinese characters. However, their error rate was significantly higher during naming Chinese characters but correlated neither with severity of phonological deficit nor with visual complexity. Their first fixation was significantly more often on the center of characters, in group C on the left. In both groups, EM variables were influenced by conspicuous features of characters, such as visual complexity, composition and structure. EM variables and scanning behavior while naming Chinese characters indicate holistic processing in the visuo-spatial pathway and were affected by conspicuous features of characters. The higher error rate in group DD could be determined by several factors, without a major role of the phonological deficit.

**Supplementary Information:**

The online version contains supplementary material available at 10.1038/s41598-024-78894-2.

## Introduction

In recent years, cross-cultural studies on reading have shown universal and specific reading mechanisms in different writing systems and that alphabetic and logographic writing systems require different skills^1–5^. The universal and specific mechanisms during reading in different writing systems have been highlighted and summarized in recent reviews^4,5^. Universal elements are meaning, phonology, print, including visuo-orthographic and visuo-motor skills. However, their weightings differ by language and writing system^4^. In transforming written language into a code for text comprehension, where word meaning is to be extracted from visual form, the interactive use of top-down and bottom-up information and general cognitive processes are universal for reading^5^. Li et al. emphasized that despite the universal processing principles, readers of different writing systems naturally adapt to a given script for efficient comprehension of a written text^5^.

Typically, in an alphabetic script, sounds are encoded at the grapheme-phoneme level, while in a logographic script, sounds are encoded at the morpheme level, and the morpheme can represent word meanings without access to spoken language^5^. In alphabetic scripts, phonological and semantic pathways are both used together, while in logographic scripts primarily the direct semantic pathway is used^5^.

Therefore, the minimum requirement for fluent reading in an alphabetic script is sufficient knowledge of a finite set of grapheme-phoneme correspondences and the ability to combine smaller units of phonemes into whole word units, which are eventually linked to semantic information about the words. This is sufficient only for regular words. For irregular and multisyllabic words some memorization of larger units, such as syllables and morphemes, as well as encoding of semantic information, are still necessary. In contrast, fluent reading in a logographic script requires sufficient knowledge of a much larger set of morphemic units, which also represent semantic information about a word, to assemble and recall the correct pronunciation of words. For alphabetic scripts, phonological abilities, especially phonological awareness^6–8^ and the phonological loop of working memory are essential for unimpaired reading^9^. Thus, a phonological deficit is considered as the major cause for developmental dyslexia (DD) in alphabetic languages like German, but not necessarily in learning to read logographic scripts, such as Chinese or Japanese Kanji. Instead, learning to read logographic scripts predominantly requires morphological, visuo-spatial and visuo-motor skills^4,5,10–16^.

There is a controversial discussion in the literature about the question, whether the phonological deficit of the children with DD in an alphabetic script also impairs learning a logographic script^1–3,17–19^.

Most children with DD in an alphabetic language have unimpaired visual skills^20–23^. Slight visual deficits are considered controversial^21,22,24,25^, and can probably only be found in subgroups^20,25,26^. Our earlier studies^20,27^ examined whether German children with DD perform better in tasks that are not letter-mediated. We presented *pictures of objects* without repetition that were displayed on lines like text, where the task was to name them without time pressure. We found that this “confrontational” picture naming was unimpaired: The children with and without DD were not statistically different from each other regarding their EM. In this previous study^20^ we analyzed the same variables as in the current one (articulation latency, number and duration of fixations). The time course of the brain activation assessed by magneto-encephalography was not different for the visual brain regions, but was delayed for language regions^27^.

This indicated that these children used the direct visual-semantic pathway and might therefore learn a logographic script more easily. For logographic scripts, the direct semantic pathway has been reported as the major processing path^5^.

The first study of this question by Rozin et al.^28^ found that children with DD, who were raised in an alphabetic writing system, can learn a logographic script easily, and that their performance while naming Chinese characters in English was comparable to that of age-matched peers.

Compared to the study of Rozin^28^, we went one step further *in a recent publication*^29^ and tested not only the meaning of a logographic character (here the German translation of the Chinese character), but also had the Chinese characters pronounced in Chinese. Our motivation was to see, whether children with dyslexia can learn a logographic script as well as children without dyslexia. Furthermore, we examined EM to receive detailed information on the children`s strategies during stimulus processing by focusing on the results of each individual participant^29^.

Regarding their EM variables, we found no significant differences between the two groups for naming pictures and naming the meaning of the Chinese characters with the German word (= German naming) and pronouncing the Chinese character in Chinese (= Chinese naming).) However, the error rate for naming Chinese characters was significantly higher in children with DD, particularly when naming them in Chinese. This result suggests that articulating Chinese characters by using the Chinese word might be impaired by their phonological deficit.

In regular alphabetic scripts, letters or syllables correspond directly to sounds (as e.g. in Finnish or German), a characteristic that is less consistently shared by alphabetic scripts with lower grapheme-phoneme-correspondence (e.g. English) and not by logographic writing systems^5,30^.

The *current report* aims to clarify the reasons why the recent research^29^ observed a higher error rate in naming Chinese characters in group DD compared with group C. Thus, if we regard the data collected in our recent publication from another perspective, we can here focus on each individual stimulus *with the following research questions*:

1. Are there conspicuous features of Chinese characters such as visual complexity (number of pixels, number and arrangement of strokes), degree of iconicity, composition and structure, e.g. containing a sub-grapheme (see below) that lead to errors and/or impaired EM variables?
2. Can the EM variables and the scanning patterns, when analyzed for each stimulus separately, provide information about the strategies during stimulus processing, especially whether Chinese characters are processed holistically (as a whole) or analytically (in segments)?
3. Are the processing strategies different between the groups?

This exploration aims to deepen the understanding of universal and specific reading mechanisms, as well as differences in behavior between children with and without DD. Here we examine the EM in response to each individual stimulus separately, to assess not only the specific main variables (articulation latencies, numbers and durations of fixations), but also the scan path on the stimulus by assessing the retinal locations used for fixating each stimulus. Reading EM have been monitored directly on the retina by a scanning laser ophthalmoscope in patients with macular degeneration in earlier studies by other authors and ourselves (e.g^31–33^). and in patients with hemianopia (e.g^34^). Our group is the only one that has used this technique on subjects with DD^20,35,36^. The purpose is to determine the absolute position of the fovea on the stimulus without requiring a calibration (see Methods). This allows a specific observation of fixation behavior. The goal was to investigate whether the Chinese characters are processed by scanning single strokes (in separate components) or as a whole (holistically).

We hypothesize that:

1. Children with DD perform worse than those without DD when naming alphabetic words in regard to their EM variables.
2. Children with DD perform similarly to the group without DD in the logographic naming tasks in regard to their EM variables.
3. Children with DD show higher error rates than those without DD when naming Chinese characters.
4. Pictures and Chinese characters are processed as a whole by the visuo-spatial pathway.
5. Naming performance does not depend on the visual complexity of Chinese characters.

We used four different naming tasks to demonstrate EM behavior in response to different stimuli as distinguished from error rate:

1. Naming alphabetic words to illustrate the difficulties encountered by children with DD in comparison with the performance during logographic tasks in the same sample of children.
2. Naming pictures of objects – as they represent a meaning by a logographic/pictographic symbol and might be processed in a similar way as the logographic Chinese characters.
3. Naming the meaning of the Chinese characters by using the German word (= “German naming”), and Pronouncing the Chinese character in Chinese (= “Chinese naming”).

## Methods

### Study design

We performed the following examinations as pre-tests on every participant during their first visit to our eye hospital: Ophthalmological examinations, reading tests, eye tracking while reading single alphabetic words and naming pictures of objects. During the subsequent school holidays, the children participated in a block of Chinese lessons (8 consecutive days, 3 h per day) at the China Center Tuebingen (CCT) - for details see paragraph “Chinese lessons”, below. During the second visit to the hospital (post-test), we performed eye tracking while the children named Chinese characters in two different tasks: Either naming the meaning of the Chinese character by using the German word (= German naming task) or pronouncing the Chinese character with the Chinese word (= Chinese naming task).) For instance, the character for “cat” can be named by the German word for cat (Katze) or the Chinese word for cat (mao).

This project was approved by the ethics committee of the University of Tuebingen medical faculty, and informed written consent was obtained from the parents and children. The research adhered to the tenets of the Declaration of Helsinki. The study was reported in an open-source online registry as a non-randomized interventional study (No. DRKS00015697; 11/10/2018), German Clinical Trials Register, Cologne, Germany.

### Participants

Most of the children with dyslexia (group DD) were recruited by the Department of Child and Adolescent Psychiatry and Psychotherapy of the University of Tuebingen, others by local child psychiatrists, by local schools, newsletter, newspaper advertisement, or by personal contacts. Out of the 409 screened children with DD, 298 had to be excluded due to additional attention deficits (140), other comorbidities, or because they lived too far away, which rendered their participation impossible. Therefore, only 111 children were eligible, but 83 of them did not respond to our invitation. Of the remaining group, 28 children with dyslexia were recruited, and after 5 of them did not show up, ultimately, 23 children with dyslexia participated in the study. Five of these missed more than 2 out of 8 lessons and had to be excluded. Therefore, all steps of the data analysis were performed on the data from 18 children with dyslexia (14 boys, 4 girls; mean age 10 years, 2.5 months; SD 11 months).

The 22 members of the control group (group C) were healthy, not reading-impaired age-matched children of 4th and 5th grade. They too, were recruited by newsletter and newspaper advertisement, in local schools and by personal contacts (10 boys, 12 girls; mean age 10 years, 2 months; SD 9 months).

The sample size calculation was performed by a software application (G*Power 3.1., RRID: SCR_013726, <www.g-power.de>) using a two-tailed model^37^. Based on our previous study^35^, the effect size was determined between the groups (Cohen’s d = 2.58) if the task was reading alphabetic words, and articulation latency was the outcome variable. With an error probability of α = 0.05 and power (1- β error probability) = 0.95, the required sample size for our study was 12 children (6 in each group). Hence, the 18 children with and the 22 children without dyslexia included in the current report clearly exceed the required sample size.

Recruitment, pretest and posttest were conducted from October 2018 to November 2019.

#### Inclusion criteria

*For whole sample*:

- 4th and 5th grade,
- Age between 9 and 11 years,
- German as native language.

Children who met the inclusion criteria were eligible for the **group DD**: Developmental dyslexia (ICD-10 F81.0) was diagnosed by specialists in child and adolescent psychiatry based on standardized test procedures according to the definition by the WHO^38^ and on the Guideline of the German Association for Child and Adolescent Psychiatry, Psychosomatics and Psychotherapy^39^, - for details see below.

Children who met the inclusion criteria were eligible for the **control group** if there was no documented problem with reading or writing skills.

#### Exclusion criteria

- Any reading impairment of other origin,
- Any disease of the eyes and visual pathway,
- Comorbidities such as ADHD, ADD, emotional disorder.

### Criteria for diagnosing developmental dyslexia (DD)

Developmental dyslexia (ICD-10 F81.0) was diagnosed by child and adolescent psychiatrists based on standardized test procedures (see below) according to the definition of the WHO^38^ and the Guideline of the German Association for Child and Adolescent Psychiatry, Psychosomatics and Psychotherapy (Deutsche Gesellschaft für Kinder und Jugendpsychiatrie, Psychosomatik und Psychotherapie e.V. (reported in English by Galuschka and Schulte-Koerne^39^).

The standardized diagnosis of DD contains a spectrum of tests. Their application in the individual case depends on the age, school grade, school type etc. The test battery always consists of an intelligence test, a reading test AND a spelling test, or a combined reading and spelling test. All children were examined by our external cooperation partners before they were included in our study. Detailed descriptions are shown in the above-mentioned Guideline^39^.

### Criteria for excluding the diagnosis “Attention deficit hyperactivity Disorder” (ADHD)

The diagnosis ADHD (ICD-10 F.90.0) was performed by child and adolescent psychiatrists and specialized psychologists based on the diagnostic criteria of the ICD 10: World Health Organization^38^ as well as the Guideline of the German Association for Child and Adolescent Psychiatry, Psychosomatics and Psychotherapy^40^.

During the diagnostic process, a detailed history, anamnesis of teachers and specific tests regarding cognitive abilities and concentration were performed, corresponding to the age of the child.

### Data at baseline

The clinical data at baseline are summarized in Table 1 and shown in detail in Table A1 in the Appendix. There were no significant differences between the groups regarding age, school grade and visual acuity. There were significantly more boys than girls in the group with DD (as in the general population). The difference between the groups was highly significant regarding their reading speed and reading errors in the Zürcher reading test.

**Table 1:** Descriptive data as median and interquartile range.

Group	Age [years] Median (IQR)	Sex	School Grade	Reading speed [wpm] Median (IQR)	Reading errors Median (IQR)	VA Median (IQR)
Group DD (*n*=18)	10.24 (9.63-10.91)	b:14, g:4	Grade 4: 9 Grade 5: 8	57.5 (45.3-78.3)	4.0 (2.0-8.3)	1.40 (1.36-1.40)
Group C (*n*=22)	10.25 (9.69-11.23)	b:10, g: 12	Grade 4: 12 Grade 5: 10	127.9 (111.2-139.5)	0.0 (0.0-0.0)	1.40 (1.36-1.40)
Between group test	TT: t(38)=-0.951, *p*=.348	χ^2^(1) = 4.310, ***p*** **= .038**, φ = -0.328	χ^2^(2) = 1.263, *p* = .532, φ = 1.78	TT: t(38)=8.556, ***p***<**.001**	MWU: U=385.000, ***p***<**.001**	MWU: U=205.000, *p*=.861

DD: Children with dyslexia (group DD), C: control (group C), g: girl, b: boy, reading speed and reading errors were assessed by Zürcher reading Test (ZLT II) in wpm: words per minute, VA: visual acuity (decimal). TT: t-test, t: t-test statistics, p=probability value, MWU: Mann-Whitney-U Test, U: Mann-Whitney-U test U value. χ^2^: Pearson’s chi-squared test, φ: Cramér’s phi. To compare the two independent groups, t-test was used for normally distributed and continuous data, Mann-Whitney U test for non-normally distributed or ordinal data, chi-square test to examine the relationship between two categorical variables.

### Examinations

#### Examinations at baseline

A complete ophthalmological and orthoptic examination was performed to exclude an ophthalmological cause for the reading difficulty. This included distance and near visual acuity, accommodation, refraction, eye position, strabismus, motility, convergence, stereopsis, saccades, eye dominance (measured by a pinhole test), pupil reaction, and the morphology of the outer and inner segments of the eye.

We measured reading performance and speed by the Zürcher reading test (ZLT II)^41^, because it provides standardized and evaluated alphabetic texts from second to sixth grade. We used sentences for the 4th and 5th grade. Reading errors were counted and reading speed was calculated as reading time in seconds divided by the sum of correctly read words, multiplied by 60.

### Eye tracking

#### Devices and experimental setup

##### Eye movement (EM) recording by Infrared Eye Tracker (IR-ET)

We used the IR-ET device “JAZZ-novo” (Ober Consulting, Poznan, Poland) to document the EM the children performed during the 4 naming tasks. The device has a spatial resolution of 0.1 degrees and a sampling rate of 1000 Hz. Correcting glasses can be placed directly on the apparatus, which prevents interference with the EM signal. The device is especially suited for use in children because of the easy set-up and minimal intrusiveness. The sound of the children’s voices was recorded on the JAZZ-novo audio track with 8000 Hz sampling frequency to document the correctness of the answers and to measure the articulation latency. A chinrest limited head movements during the presentation of stimuli on a 21-inch CRT monitor at 25 cm (10 inch) distance.

The following EM variables were the main outcome measures:

- *Articulation latency* as a measure of naming speed, defined as the time between the end of the first saccade to the stimulus and the beginning of articulation (assessed by the visible signal on the audio track). Since articulation latency was measured during eye tracking, we include these data in the EM variables.
- *Number of fixations* to assess the degree of difficulty while the child examined the stimulus until beginning of articulation. A fixation was defined as a period without eye movements of = > 0.5° that was followed or preceded by a saccade and was at least 100 ms long.
- *Fixation duration* as a measure of information intake, including processing time. Fixation duration per item was calculated (neglecting saccade duration) as $fixation\,duration\,\left[s\right]=\frac{articulation\,latency\,\left[s\right]}{number\,of\,fixations}$.

Before the examination, a short calibration procedure required making saccades between crosses in the corners of the screen.

Before the appearance of each stimulus, the children fixated a cross (size = 0.8°) that was presented 5° to the left of the center. The stimulus then appeared in the center to trigger a saccade with a defined target position. This allowed exact determination of the beginning of the first fixation on the stimulus. The latter procedure was also applied during EM recording by SLO.

##### Eye movement recording by scanning laser ophthalmoscope (SLO)

**Fig. 1:**
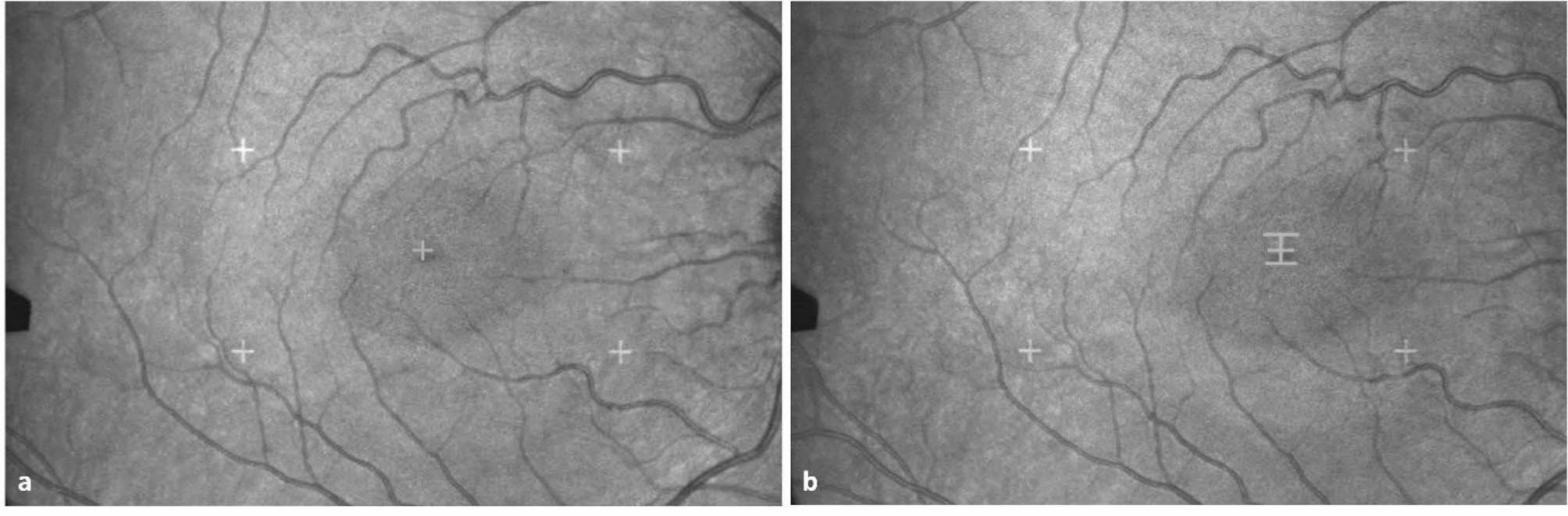
A picture of the retina during examination by the Scanning Laser Ophthalmoscope (SLO). The retina and the stimuli are displayed simultaneously in real time. Due to the optics of the device, the examiner sees the image and the stimulus upside down, but the participant sees the stimulus upright (white on a red background). (**a**) Retinal image of a right eye with foveola (the center of the fovea) fixating a small fixation cross in the center and 4 crosses in the periphery (20 × 10° window of interest, to present the relative size ratio). The horizontal crosses were 16.1° apart and the vertical ones 8.8°) (**b**) Retinal image with the fovea fixating a Chinese character, here “king” (upside down).

A scanning laser ophthalmoscope (SLO 101, Rodenstock Instruments, Ottobrunn, Germany) was used to assess the same EM variables as with the IR-ET. However, this instrument visualizes the retinal locations that were used to fixate each stimulus. This is possible, because the SLO scans each stimulus directly onto the retina, so that it is visible at that retinal location on a video monitor (Fig. 1). This image continuously shows the absolute position of the center of the fovea relative to the stimulus position. The SLO has a temporal resolution of 25 Hz (50 half-frames/s) and a spatial resolution of 0.1 degrees. As it measures the EM only monocularly, we recorded all movements of the dominant eye during the whole experiment while the fellow eye was covered.

### Analysis of the SLO data

The SLO is especially suited for illustrating details of fixation behavior on the stimulus, which cannot be seen on the IR-ET recording, because it does not show an image of the retina (see above).

We used the following procedure to determine the main EM variables and the fixation location: The digital video sequences from the SLO were analyzed to detect retinal movements using custom-designed motion tracking software that automatically tracks the position of a user-defined landmark, e.g. a retinal vessel branching. Recordings with mechanical artifacts caused by instability of head or eye position, could not be analyzed regarding their scan paths in 4 of 168 (2.38%) alphabetic words in group C and in 8 of 128 (6.25%) in group DD (Fisher`s exact test *p* = .083); in 1 of 62 (1.61%) pictures in group C and in 5 of 47 (10.6%) pictures in group DD (*p* = .052) and in 26 of 231 (11.26%) Chinese characters in group C and in 24 of 135 (17.78%) Chinese characters in group DD (*p* = .057). Thus, these few episodes were deleted.

While these artefacts were observed more frequently in group DD, the differences between groups DD and C were not significant.

For all 4 naming tasks, single stimuli were presented during EM recording by **IR-ET** and by **SLO** for quantitative analysis, and for visualizing the EM scan paths on the stimulus. The scan paths showed the sequence of fixations and the saccades (amplitude of = > 0.5°) between them, while the participant examined the stimulus until the beginning of articulation (Figs. 3, 4, 5, 7, 9, 10, 11, 13, 14, 15 and 16).

### Stimuli

We applied 3 different stimulus types: Alphabetic words, pictures of objects, and Chinese characters. The instructions, given in German language, were:

First look at the fixation cross. When the stimulus appears, look at it and name it as soon as possible.

The stimuli were presented during 4 different tasks in separate blocks:

1. Read the German word aloud.
2. Say in German what the picture shows.
3. Say the meaning of the Chinese character in German (= German naming task).
4. Say the meaning of the Chinese character by the Chinese word (= Chinese naming task).

All stimuli were presented only once, except for the Chinese characters for cat (G11), pig (G12) and rat (G6) – for details see below.

#### Alphabetic words

**Fig. 2:**
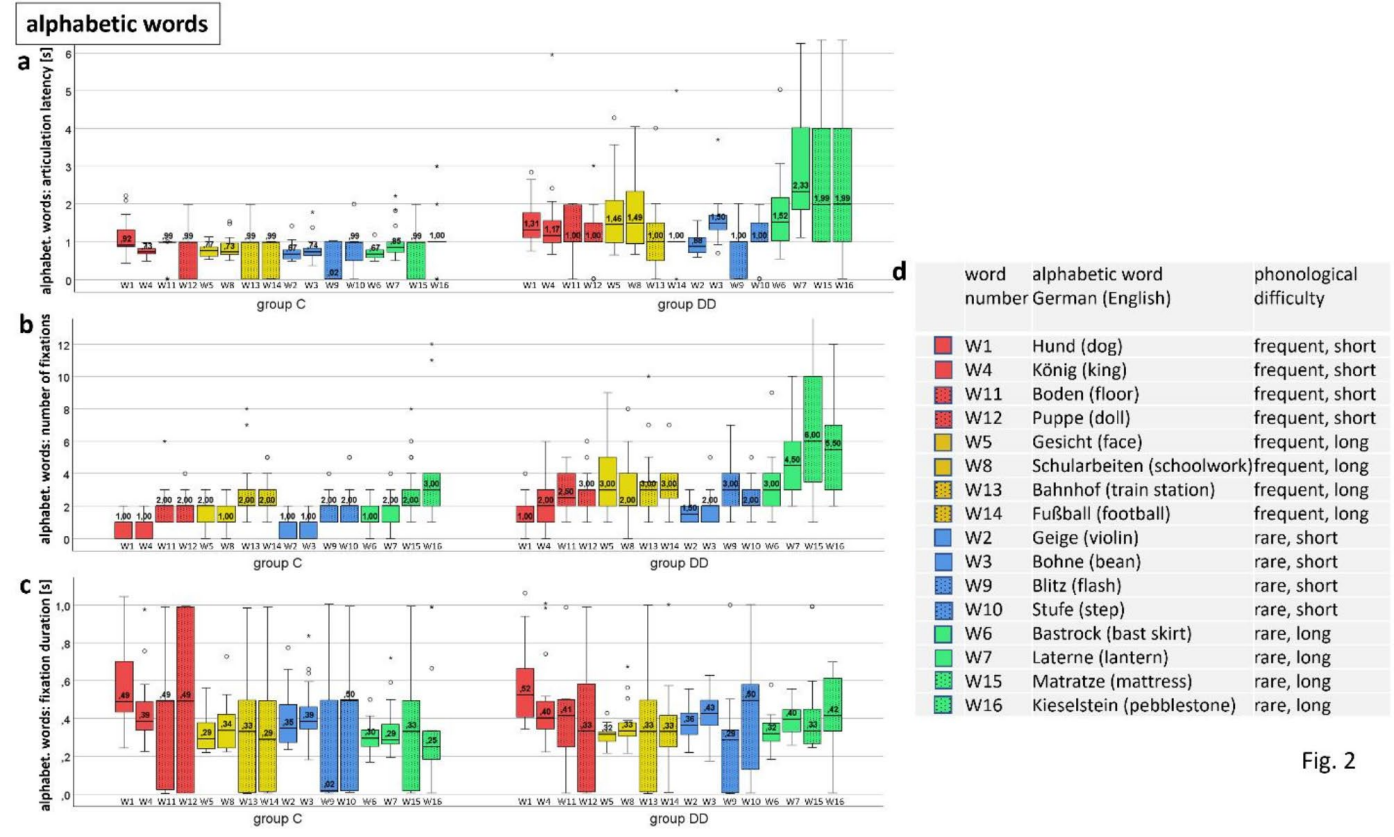
Reading alphabetic words with increasing phonological difficulty. *Medians of EM variables of each of the 16 single words* assessed by IR-ET (W1-W8; plain color) and by SLO (W9-W16, punctate color). Words of the same phonological difficulty are displayed in the same colour. (**a**) *Articulation latency* increased markedly in group DD with increasing phonological difficulty, - *significantly different for all words except W9 (flash)***–** see Table 2. (**b**) The *number of fixations* increased slightly with increasing phonological difficulty in group C, and markedly in group DD, especially for rare long words (W6, W7, W15, W16). The difference was significant for all words except W1 and W11 (short frequent words), W13 (train station) and W14 (football) – both very frequent long words. (**c**) There was no significant difference of *fixation durations* between the groups - except for W 7 (lantern, rare long). (**d**) Characteristics of the words. It is evident that the children with DD increase mainly the number of fixations with increasing phonological difficulty. See also Figs. 3, 4, 5 and Table 2.

Sixteen single words of different phonological difficulty were presented during EM recording, 8 of them with the IR-ET and 8 with the SLO (see Fig. 2d). The phonological difficulty was derived from word length (short: 4–5 letters vs. long: 7–12 letters) and from the frequency in the German vocabulary. The words were taken from the basic vocabulary required for elementary school, which were extracted from 800 written and 2,400 oral essays of elementary school children^42^. The words had already been used on different children in our previous study^35^. They were set in Times New Roman at a font size of 14 pt. At a viewing distance of 25 cm, the lower-case letter “n” was 0.5° wide. Figure 2d shows the words with increasing phonological difficulty.

#### Single pictures

Picture naming is a non-letter-mediated task that can be solved by visual processing with direct access to the meaning. In this regard, it can be considered similar to naming logographic Chinese characters.

Nine single pictures were presented for quantitative analysis, 6 with the IR-ET, and 3 with the SLO to visualize the scanpaths.

In this task, the children were asked to name the single pictures of objects in German without time pressure (confrontational naming, CN) (Fig. 6d). The size of the pictures differed in horizontal and vertical extent between 1.8° and 4.4° (the horizontal extent of the picture “duck”). The size of the pictures was adapted to the size of the Chinese characters to preserve the original aspect ratio of their outlines. The main criteria for the selection of pictures were easy recognizability and familiarity of objects to children. The answers were considered correct if the child named the correct or an umbrella term (For instance, the answer “fish” was accepted as correct for the picture “shark”). The pictures were taken from the same list as in our previous study^20^.

#### Chinese characters

Twenty-four single characters were presented during EM recordings, 12 with the IR-ET and 12 with the SLO (see Fig. 8d and 12d). Four separate blocks of characters were presented, each of them with increasing visual complexity: IR-ET: German naming: G1-G6, Chinese naming C1-C6, SLO: G7-G12, C7-C12. For each trial the participants saw only one stimulus. The characters 猫 (mao, cat), 猪 (zhū, pig) and 鼠 (shǔ rat) occurred twice: The characters cat and pig had to be named during SLO-recording with the German word and during IR-ET with the Chinese word. For rat it was vice versa. Thus, we could differentiate between recognizing the meaning (German naming) and remembering the Chinese word/articulation (Chinese naming).

In Table A2, **all 24** characters presented during EM recording are shown with increasing visual complexity, which was the same order, in which they were presented to the children. Visual complexity was defined by the number of pixels after Sun et al.^43^: To calculate character complexity, the authors generated PNG image files for each character (font: SimHei, font size: 80, font color: black, image background: white) and defined the visual complexity of a character as the number of non-white pixels in these image files. The one-character Chinese words were selected from a storybook about the Chinese Zodiac that was used as the basis for the lessons (see below). Twelve single characters were to be named in German (Figs. 8, 9, 10 and 11), and 12 in Chinese (Figs. 12, 13, 14, 15 and 16).

The Chinese characters were set in the smallest legible font size. We did not use typefaces in calligraphic styles to avoid using unnecessary pixels and to make character recognition easier. The characters were presented in the font SimHei (26 pt), corresponding to 1.8° (width and height) at a viewing distance of 25 cm. Font size and typeface were decided after a pre-test with native speakers from the China Center Tuebingen (CCT) and the Department of Quantitative Linguistics at the University of Tuebingen. A dummy experiment was performed before data collection on two native speakers of Mandarin Chinese.

Conspicuous features of characters were visual complexity (number of pixels, number and arrangement of strokes), degree of iconicity, structure and composition (e.g. containing a sub-grapheme). Several characters, e.g. “„cat“,”„pig“,”„monkey“, contain the same or similar elements, the sub-grapheme, on the left, which means “wild animal“- see Table A2 in the Appendix- causing complex challenges (see Discussion).

#### Percentage of correct answers

The percentage of correct answers for each stimulus was determined by having a Chinese teacher and a native speaker of Chinese listen to the soundtrack.

### Chinese lessons

The Chinese classes were conducted for three hours each on eight consecutive days; the teacher (author VS, a German expert of sinology) was supported by a native Chinese speaker. The children with and without DD were taught together in groups of 6–8 participants, while the teachers were not told which of the children had been diagnosed with DD. Therefore, there was no difference between the amount of instruction between the two groups. The materials and the didactic concept were developed by author VS at the China Center Tuebingen. Central criteria for the Chinese characters taught in class included visual complexity, degree of iconicity, composition and structure, similarity or dissimilarity, familiarity of the meaning as well as phonetics. A total of 37 characters were selected to be taught in the lessons in cooperation with the Department of Linguistics (author CCS). Their order is shown in Table A3 in the Appendix. In each lesson, on average 5 new items were taught. The decision about the selection of characters was guided by the important didactic goal that the characters should be embedded, both, within the culture and in authentic language interactions. The thematic frame was the story of the origin of the Chinese zodiac (based on the picture book *“The Imperial Race”*; in German: *“Das kaiserliche Wettrennen”)*^44^. Methods of discovery-based learning, visual integrative approach, and cooperative learning were applied. The use of letters in print was consistently avoided during the course to compensate for possible phonological deficits: Neither the romanization system *Hanyu Pinyin* (a phonemic transcription of the Chinese language) was used, nor was the German meaning of the Chinese characters spelled in letters. The pronunciation of the characters was taught and provided by the teacher. The pronunciation of a character was accepted as correct if it was intelligible and if the initial and final sounds were approximately correct (with a certain tolerance for difficult initials that do not exist in German). As the children were absolute beginners, we did not include the four tones in the analysis.

We examined only single characters. McBride et al.^4^ found in non-reading impaired Chinese children that individual characters were more difficult to identify than 2-character words. Their identification requires excellent visuo-orthographic skills and exact memorization^4^. Visuo-orthographic memorization is therefore a focus of teaching character recognition and memorization^4^. This aspect was also considered during the lessons in our study.

### Learning App

All participants were provided with a specially created learning app in order to support memorization and individual learning – also without using any letters. The app was used **after** the end of the total block of lessons. The app showed only a picture of the Chinese character’s meaning. Clicking the button on a character played the correct Chinese pronunciation (see below for details). The app (for Windows and MacOS) was developed for the use at home after the block of lessons was finished. The software was provided via a download link. If the child’s family did not have access to a computer, we loaned them a notebook computer with the preinstalled app to take home.

We provided the learning app to bridge the gap between the last lesson and the examinations at T2, which could be of individually different length due to availability of the children. This was supposed to give the children the chance of not to forget, what they had learned in class. In the app, 36 Chinese characters that had been taught before, during the block of lessons, were arranged in 4 columns on the left and in 9 rows. The characters were presented in the simple SimHei font (size = 40pt), which uses plain strokes without serifs. Each character could be clicked by the mouse, and a picture illustrating the meaning of the character appeared on the right side, while the computer pronounced the Chinese character through the audio output of the computer. The pronunciation was the same as it would have been in Pinyin.

Each time the app was restarted for use at home, the characters were placed in a different position on the panel. That way, the characters were not just memorized by their order of appearance on the screen.

### Statistical methods

All statistical analyses were performed by SPSS statistics software (IBM Corp. Release 2021, Version 28.0. Armonk, NY). We used the Shapiro-Wilk normality test and graphical Q-Q plot*s* to detect deviations from normal distributions. For confirmatory statistics we used the non-parametric Mann-Whitney U-test for between group comparisons, as most of the data were not normally distributed, so that descriptive data are reported as medians and interquartile ranges (IQR). Boxplots showing the results for each stimulus (pictures, alphabetic words, and Chinese characters) are presented by pairwise exclusion (<https://libguides.library.kent.edu/SPSS/Explore>). We consider all comparisons between groups separately and independently of each character and, thus, we did not perform a multiple comparison test. The level of significance α was set to 0.05 (two-sided).

## Results

Whenever we use the term “significant”, we mean “statistically significant”. In the text, we will mention only the significant values, while all other calculations are shown in the tables.

### EM responses to individual stimuli

The main EM variables (articulation latency, number of fixations, fixation duration) are shown in Figs. 2, 6, 8 and 12. Those for the fixation locations on each stimulus are displayed in Figs. 3, 4, 5, 7, 9, 10, 11, 13, 14, 15 and 16. The statistical values are reported in Tables 2, 3, 4 and 5.

#### Reading alphabetic words

##### Analysis of main variables during EM recording by IR-ET and SLO while naming alphabetic words

**Articulation latency** (Fig. 2a) increases slightly with increasing phonological difficulty in group C and markedly in group DD, especially in rare long words (W6,W7,W15,W16). All words (except W9, flash) showed highly significant differences between the groups – see Table 2.

The **number of fixations** (Fig. 2b) increases in group C only slightly and only for rare long words, but markedly with increasing phonological difficulty in group DD - already for short words, but especially for rare long words. The difference between the groups was significant for all words except 4 very frequent words (W1, W11, W13, W14) – see Table 2.

**Fixation duration** (Fig. 2c) was not changed by increasing phonological difficulty and did not differ significantly between the groups except for W7 (rare long), which was longer in group DD.

**Table 2:**
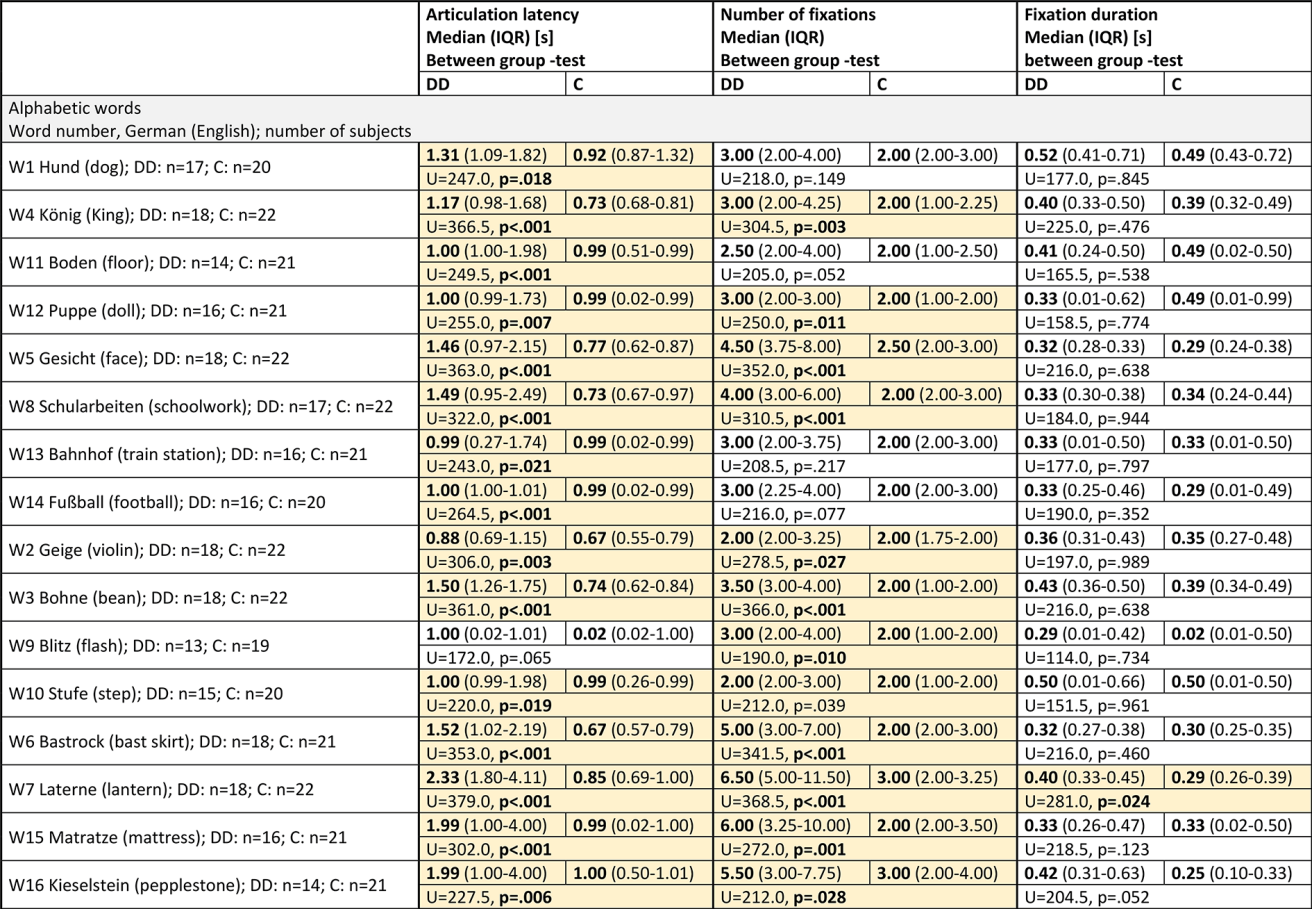
Alphabetic words.

DD= children with dyslexia, C= control group, n= number of subjects, IQR= interquartile range, U= Mann-Whitney U-test statistics, U-value, p= probability value, s= seconds, between-group-test= Mann-Whitney U-test. The order of the words in the table are shown according to increasing phonological difficulty (frequent short, frequent long, rare short, rare long). The word number is shown according to the order of presentation. The yellow fields highlight the significantly different results.

#### Scan paths during EM recording by SLO

General remarks regarding SLO-figures showing scan paths (Figs. 3, 4, 5, 7, 9, 10, 11, 13, 14, 15 and 16).

The figures show examples of scan paths during performing the different tasks that reflect frequently occurring behaviors. The image of the retina together with the stimulus is displayed upside down, but the enlarged inset shows the stimulus upright, as it was seen by the child.

The images show the scan paths during examining and naming the stimuli, where the dots show the fixations and the lines the saccades between them.

The time course is shown by the changing color of the points (dots) and lines - from early (black) to dark red and increasingly lighter red.

#### Scan paths during EM recording by SLO while naming alphabetic words

**Fig. 3:**
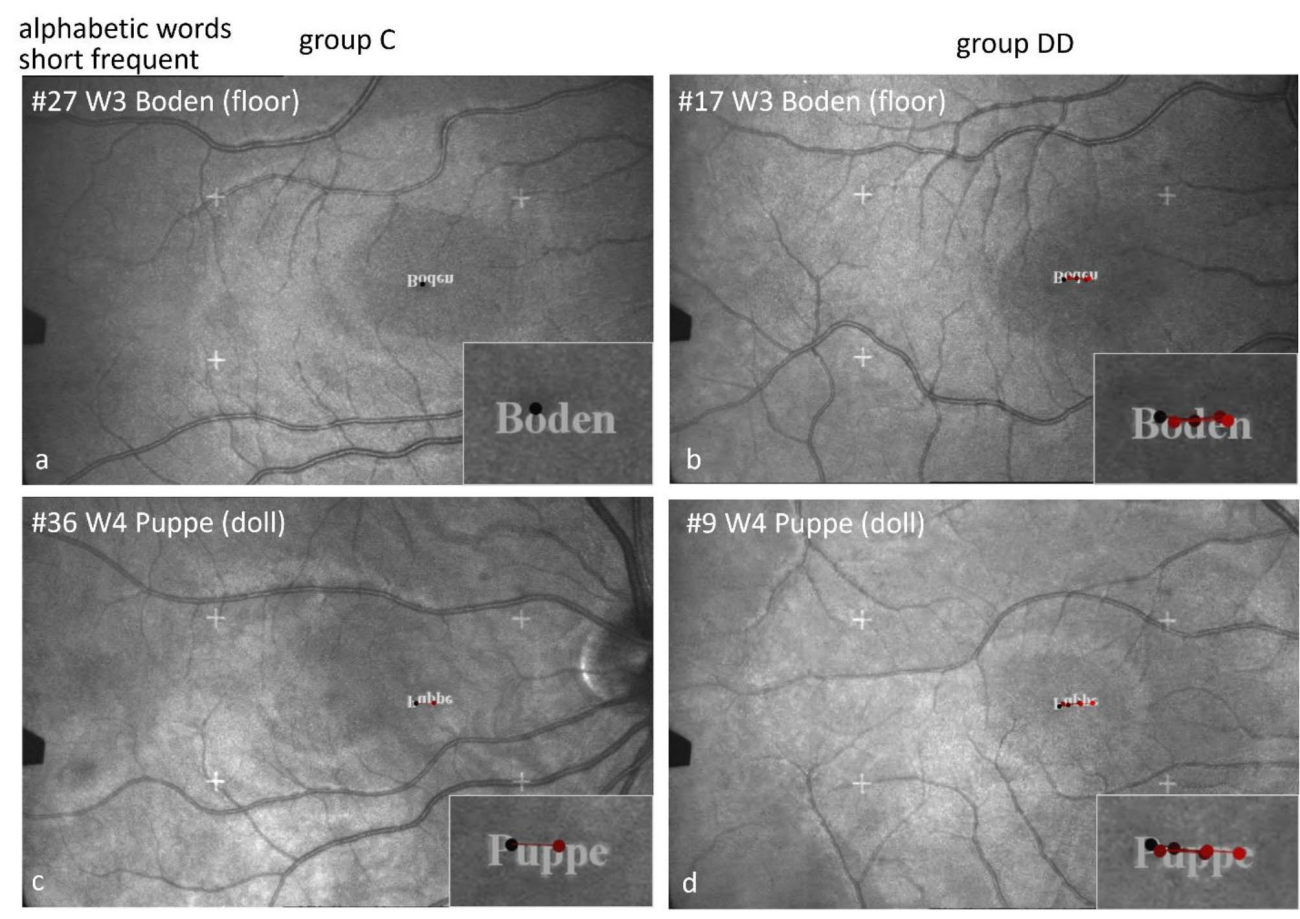
Individual SLO scan paths when reading frequent short words. The difference between the groups is present already in frequent short words: the child of group C (**a**) names the word “Boden” (floor) after one fixation, the child of group DD after 5 fixations (**b**).

**Fig. 4:**
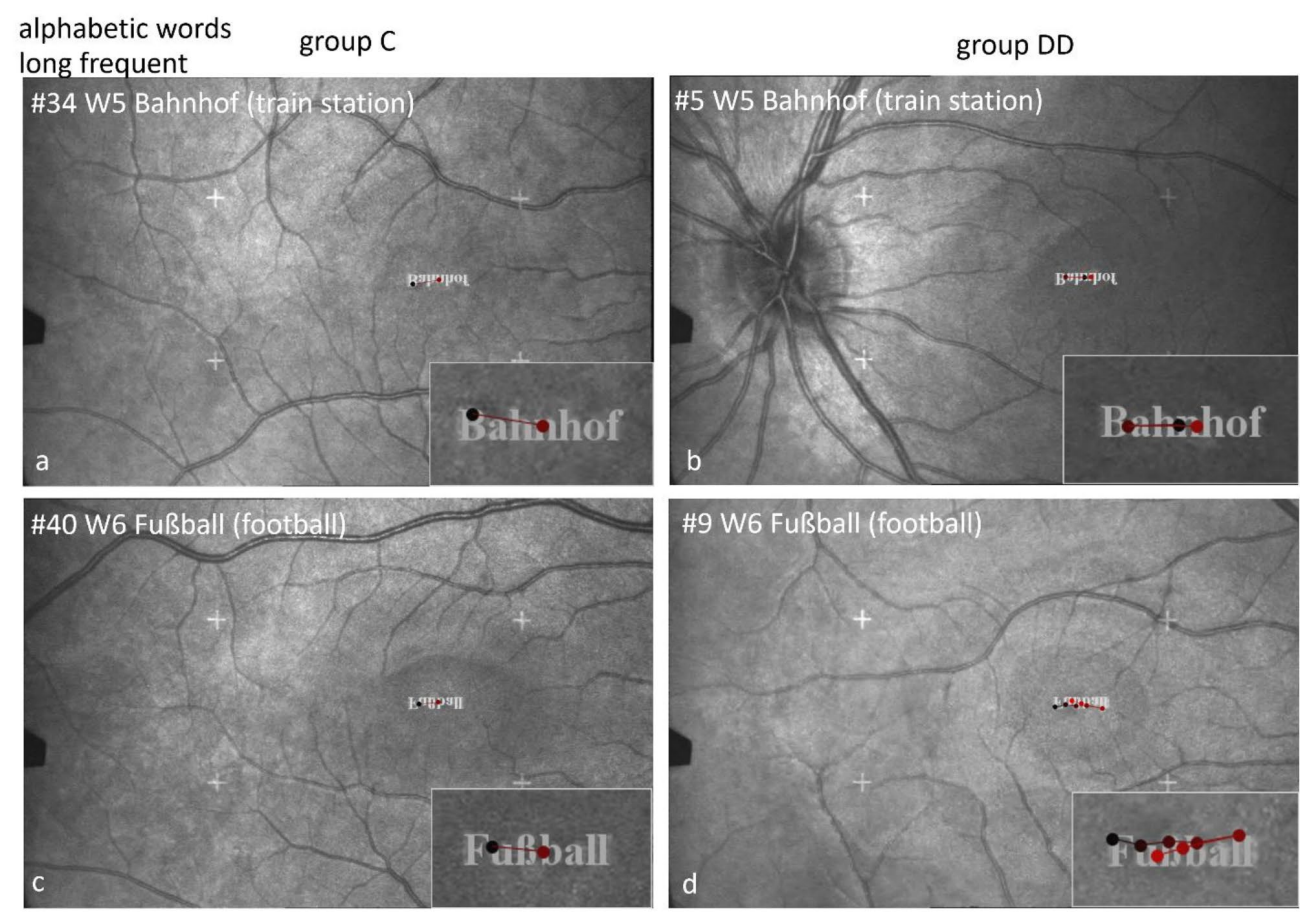
Individual scan paths measured by SLO while reading frequent long words. The children without DD make 2 fixations (**a**, **c**), those with DD either 3 (**b**) or more fixations (**d**).

**Fig. 5:**
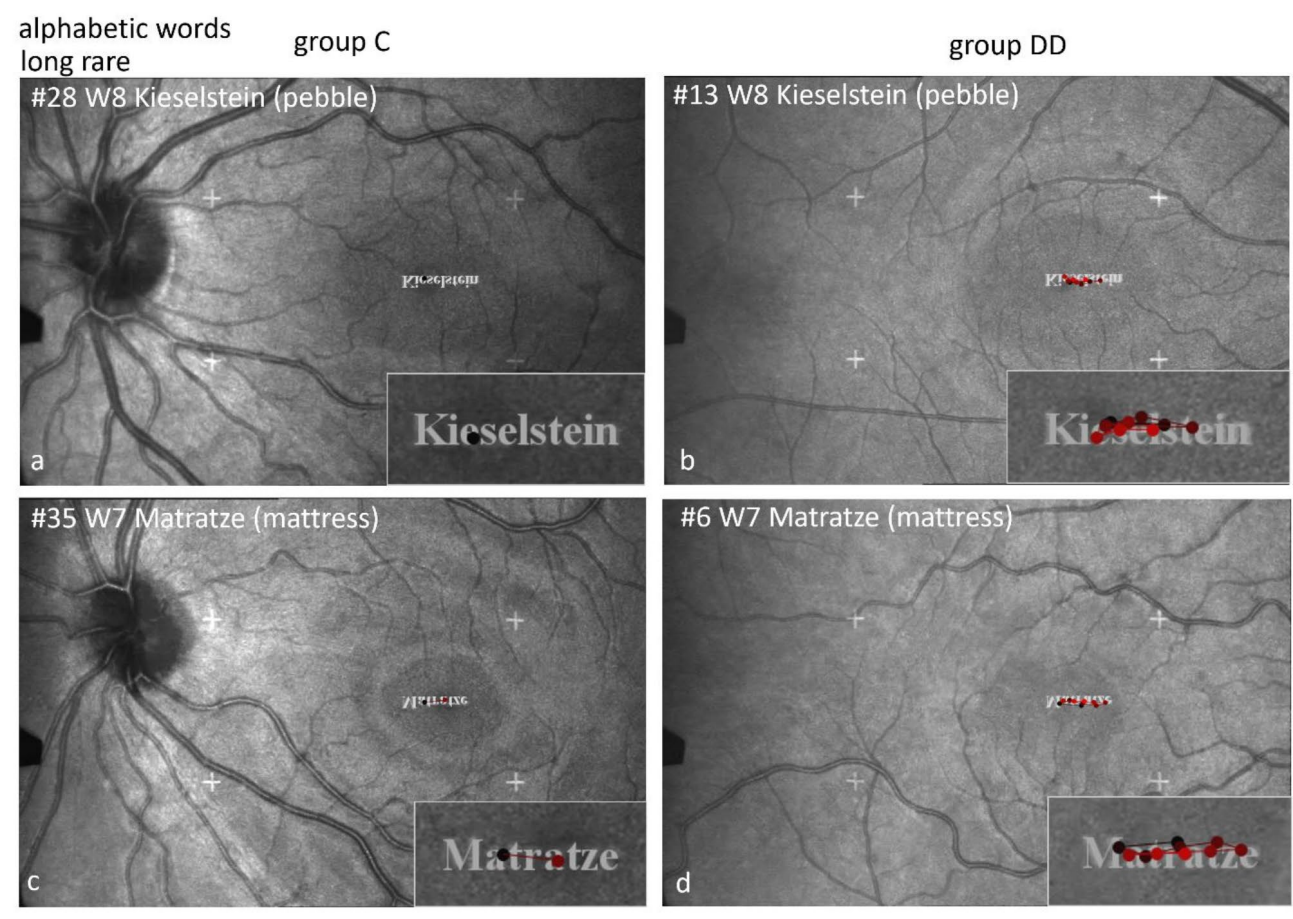
Individual scan paths measured by SLO when reading rare long words. An extreme example is the difference between the child of group C with only 1 fixation on the rare long word “Kieselstein” (**a**) compared with the child of group DD with numerous fixations (**b**).

Figures 3, 4 and 5 show examples of scan paths of individual children while reading alphabetic words that demonstrate the differences between the groups. The left columns in Figs. 3, 4 and 5 show two examples of children from group C, the right columns those of group DD. The words are displayed with increasing phonological difficulty. The number of fixations rises much more prominently with increasing phonological difficulty in group DD than in group C.

Figure 5 displays the marked difference between the groups when reading rare long words. It is evident that the number of fixations increases with increasing phonological difficulty much more in children with DD than in controls.

#### Naming pictures

##### Quantitative analysis during EM recording while naming pictures

**Fig. 6:**
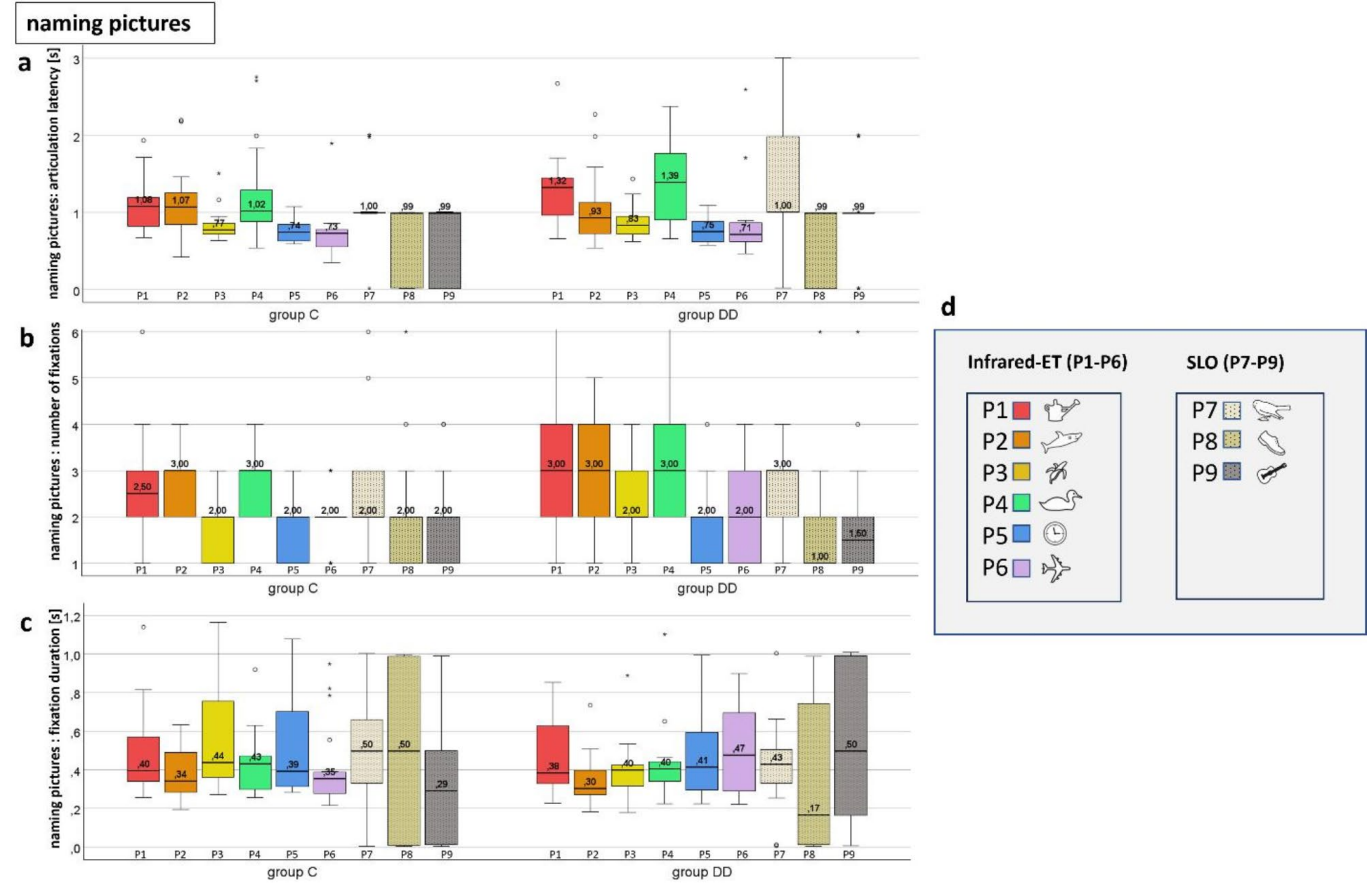
Naming pictures. (**a**) Articulation latency, (**b**) number of fixations, (**c**) fixation duration, (**d**) stimuli. Medians of the EM variables of each of the 9 single pictures assessed by IR -ET (P1-6) and by SLO (P7-9). There were no significant differences between the groups, except for a higher number of fixations for P3 (banana) in group DD, which was compensated by a shorter fixation duration. See also Fig. 7 and Table 3.

**Articulation latency** (Fig. 6a), **number of fixations** (Fig. 6b), **and fixation duration** (Fig. 6c) did not show significant differences between the groups except for P3 (banana) in number of fixations (more in group DD, *p* = .008), and fixation duration, which was shorter in group DD (*p* = .049). The increased number of fixations for P3 is compensated by a shorter fixation duration, resulting in a normal articulation latency – see also Table 3.

**Table 3:**
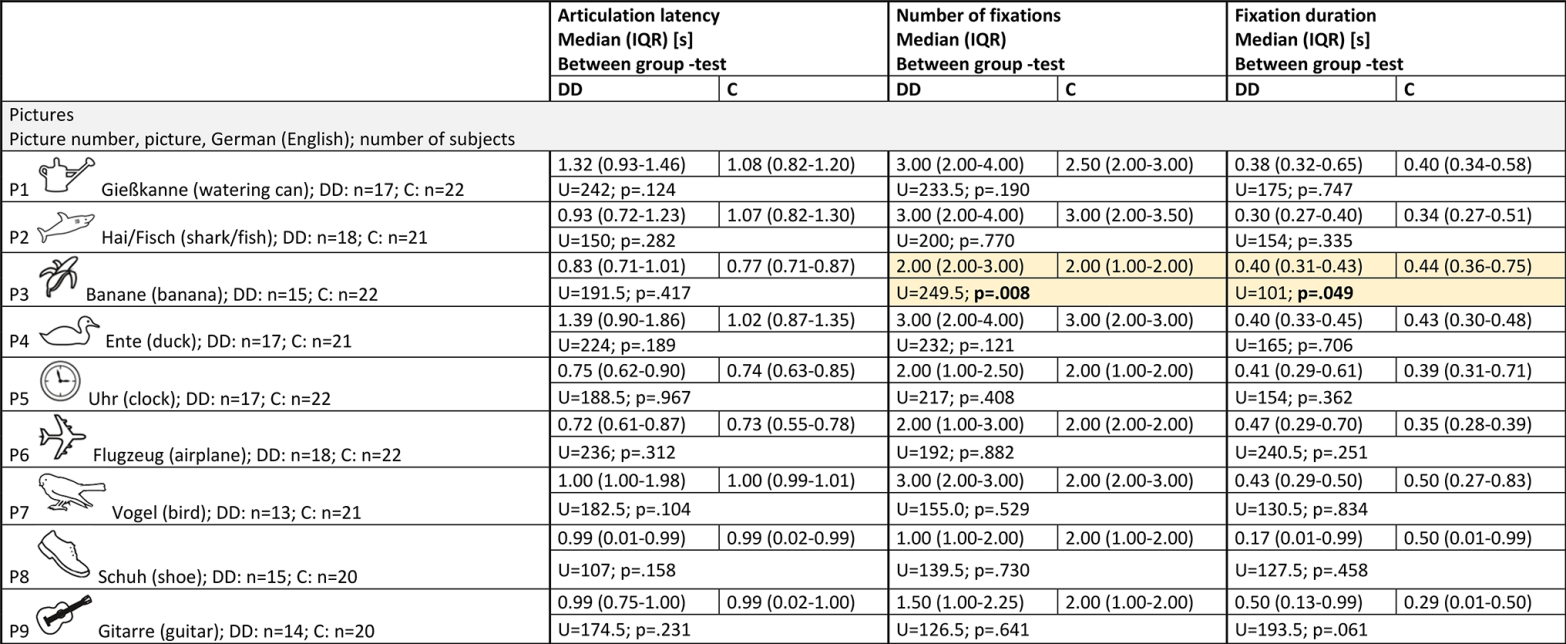
Pictures.

DD= children with dyslexia, C= control group, n= number of subjects, IQR= interquartile range, U= Mann-Whitney U-test statistics U-value, p= probability value, s= seconds, between-group-test= Mann-Whitney U-test. The yellow fields highlight the significantly different results.

##### Scan paths during EM recording by SLO while naming pictures

**Fig. 7:**
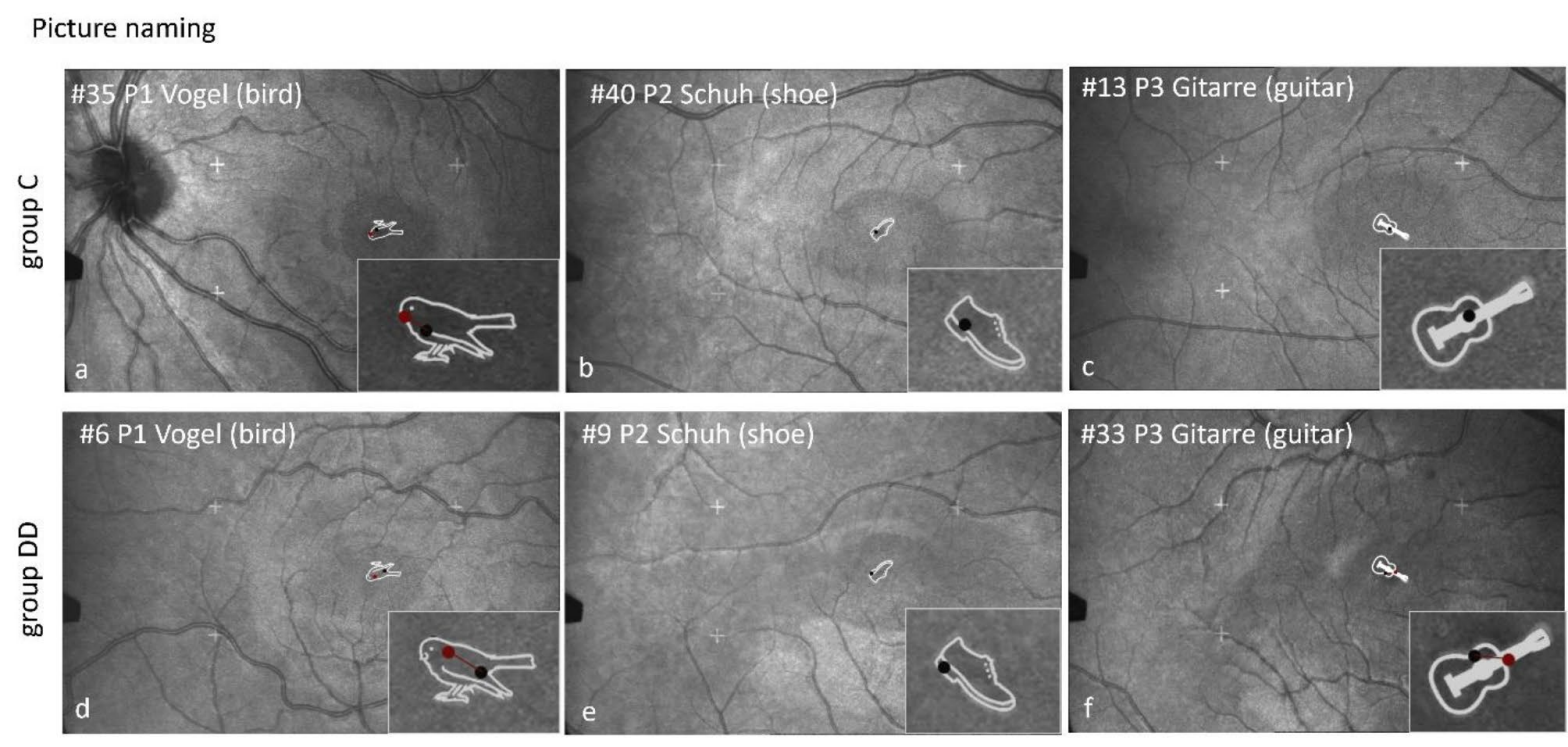
Individual SLO scan-paths during picture naming. The upper row shows 3 examples of 3 children of group C, the lower row 3 examples of group DD. It is evident that the scan paths are similar between the groups when naming pictures, as they show only 1–2 fixations per picture.

Figure 7 shows scan paths while naming pictures. The examples show children in both groups with 1 or 2 fixations on the picture. For details see Table 3.

#### Naming Chinese characters

Altogether twenty-four individual Chinese characters were presented. The EM elicited by 12 of them were measured by IR-ET and 12 by SLO. Twelve were to be named in German (Figs. 8, 9, 10 and 11), and 12 in Chinese (Figs. 12, 13, 14, 15 and 16). Their presentation was ordered by increasing visual complexity.

### Naming Chinese characters in German

#### Quantitative analysis during EM recording while naming Chinese characters in German

**Fig. 8:**
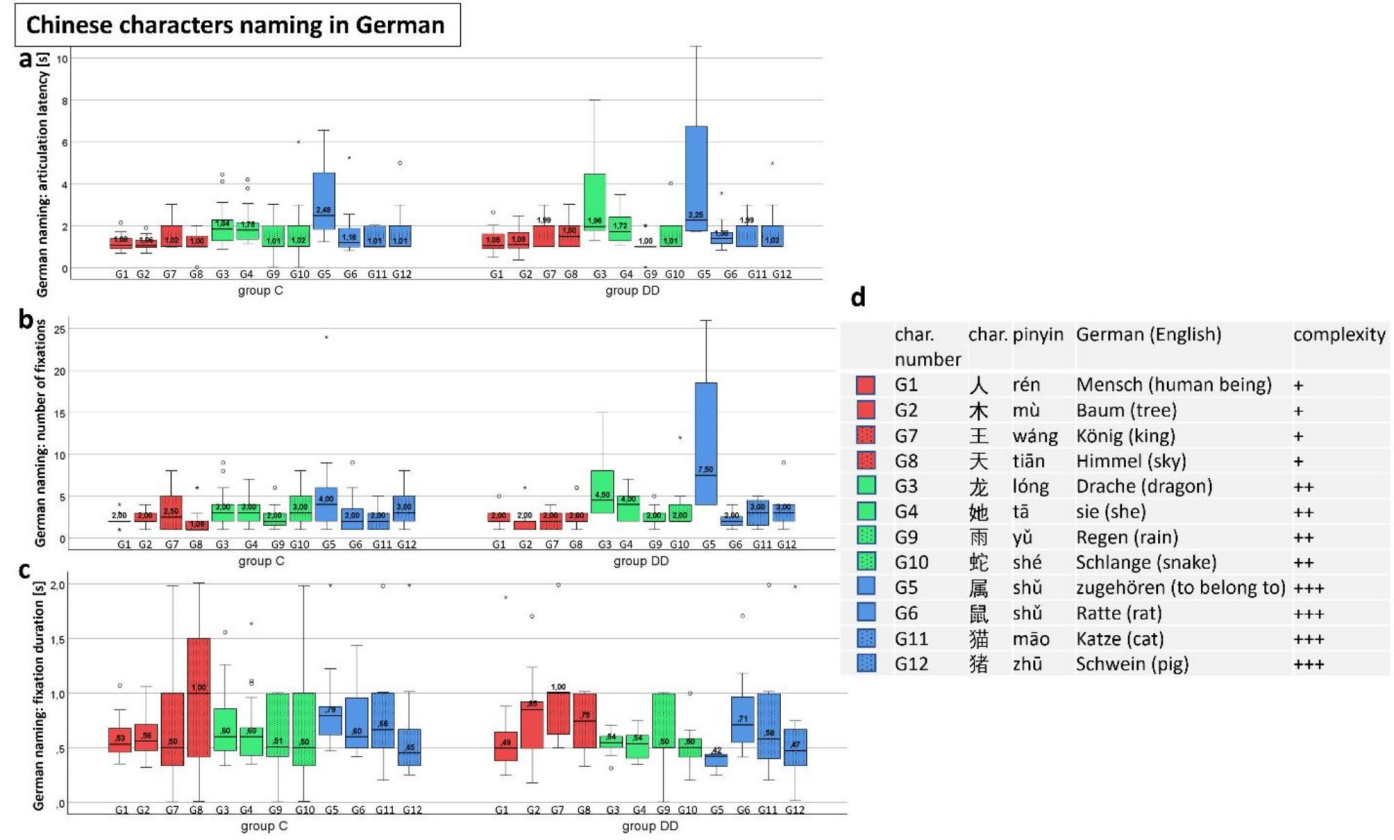
Naming Chinese characters in German. (**a**) Articulation latency, (**b**) number of fixations, (**c**) fixation duration, (**d**) Chinese characters with increasing visual complexity − 6 by IR-ET (G1-G6, plain colour ) and 6 by SLO ( G7-G12, punctate colour). Characters of the same visual complexity are displayed in the same colour: For most stimuli, all three variables did not differ significantly between the two groups - except for G11 in articulation latency (longer in group DD) and for G3 for the number of fixations (higher in group DD) and for G5 for fixation duration (*shorter* in group DD). Visual complexity does not play a major role. See also Figs. 9, 10, 11 and Table 4.

**Articulation latency** (Fig. 8a) did not increase with increasing visual complexity in either group, but individual characters were named after a longer articulation latency in both groups. Especially noticeable is the long articulation latency for the character 属 “to belong to” (G5) in both groups and the character 龙 dragon (G3) in group DD, without significant difference between the groups. A significantly longer articulation latency was found for character 猫 mao (cat) (G11) in group DD – see Table 4.

**The number of fixations** (Fig. 8b) produced a similar result: Particular characters required a markedly higher number of fixations to be named. A significantly higher number was found for 龙 dragon (G3) in group DD. Visual complexity showed a moderate, significant correlation with the number of fixations in group DD (see Table 4 and below paragraph “visual complexity”).

**The fixation duration** (Fig. 8c; Table 4) did not depend on visual complexity either. The values for both groups were quite similar - except for character 属 (“to belong to” G5), which was shorter for group DD. However, only 4 children of group DD named the character correctly – in contrast to 15 children of group C (see Discussion for details). This might indicate that group DD lengthened their articulation latency in “difficult” characters by increasing the number of fixations, whereas group C tended to lengthen fixation duration (see Table 4).

**Table 4:**
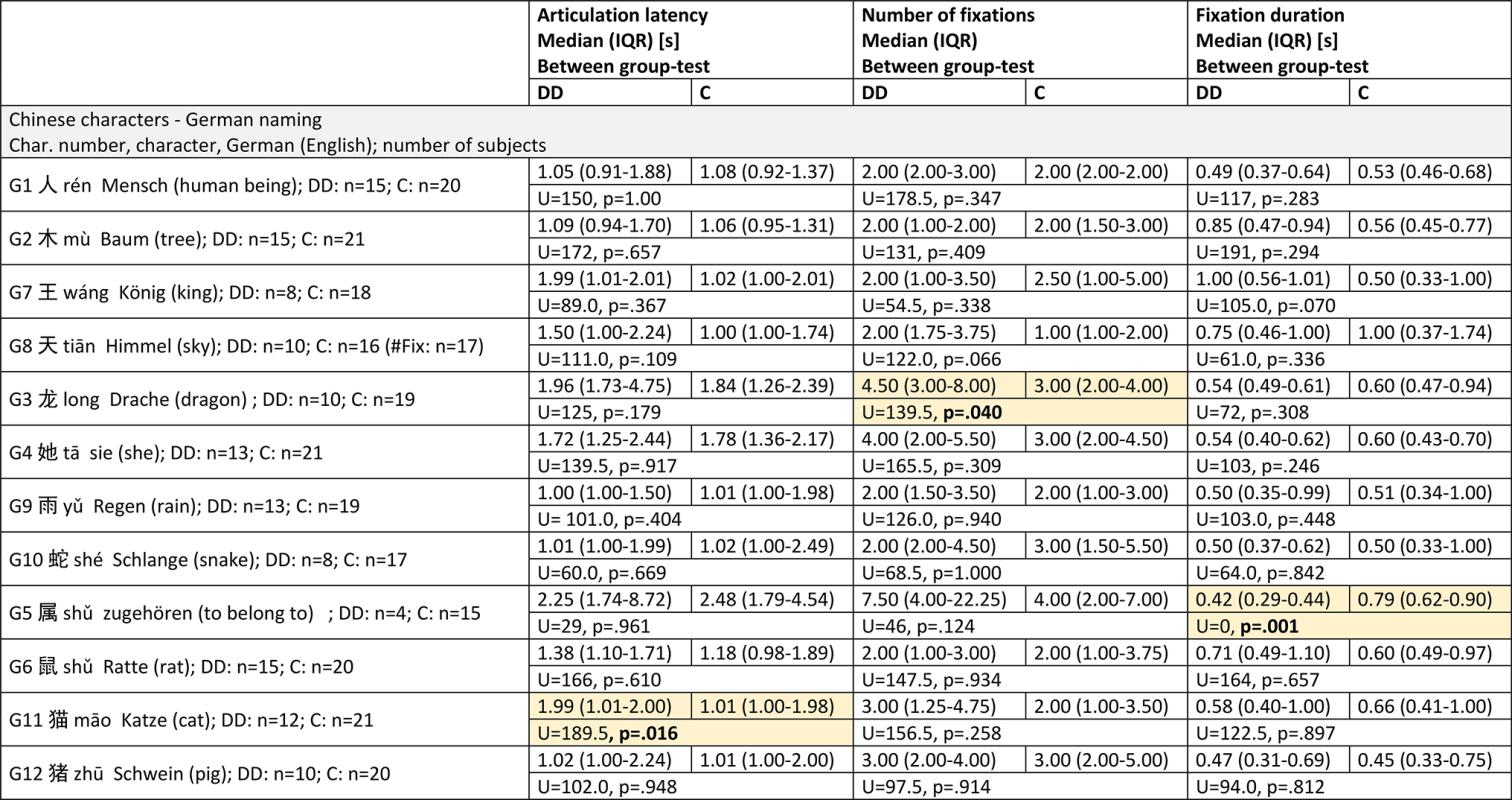
Chinese characters, German naming.

DD= children with dyslexia, C= control group, n= number of subjects, IQR= interquartile range, U= Mann-Whitney U-test statistics U-value, p= probability value, s= seconds, between-group-test= Mann-Whitney U-test. The characters were presented with increasing visual complexity, G1-G6 with the Infrared Eye Tracker, G7-G12 with the SLO. The yellow fields highlight the significantly different results.

#### Scan paths shown by EM recordings with the SLO while naming Chinese characters in German

**Fig. 9:**
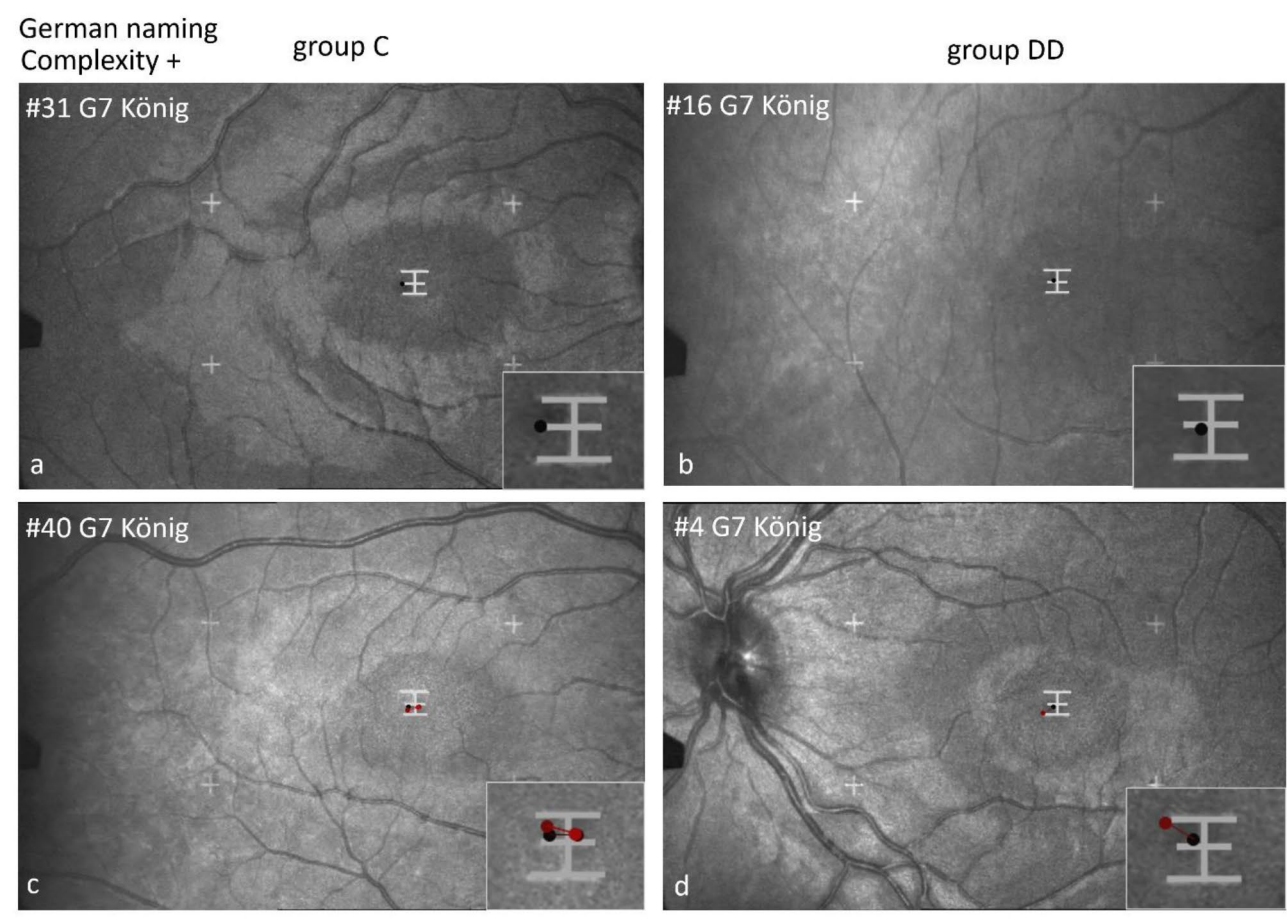
Individual scan-paths when naming Chinese characters of low visual complexity in German. Two examples from each group (group C left, group DD right) are shown per character, in the upper row with 1 fixation, in the lower row with 2–3 fixations.

**Fig. 10:**
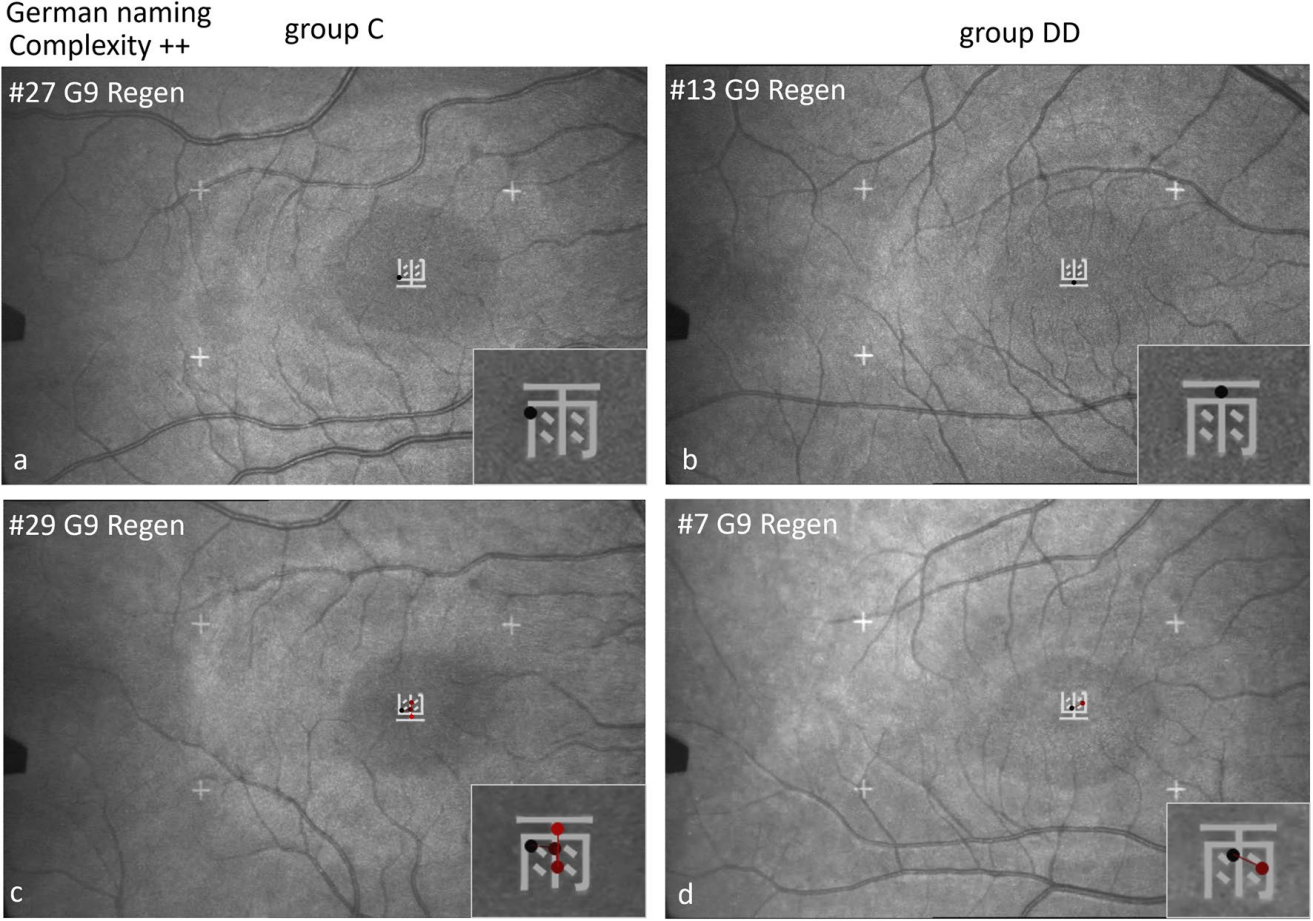
Individual scan paths when naming Chinese characters of medium visual complexity in German. In the upper row with 1 fixation, in the lower row with 2–4 fixations.

**Fig. 11:**
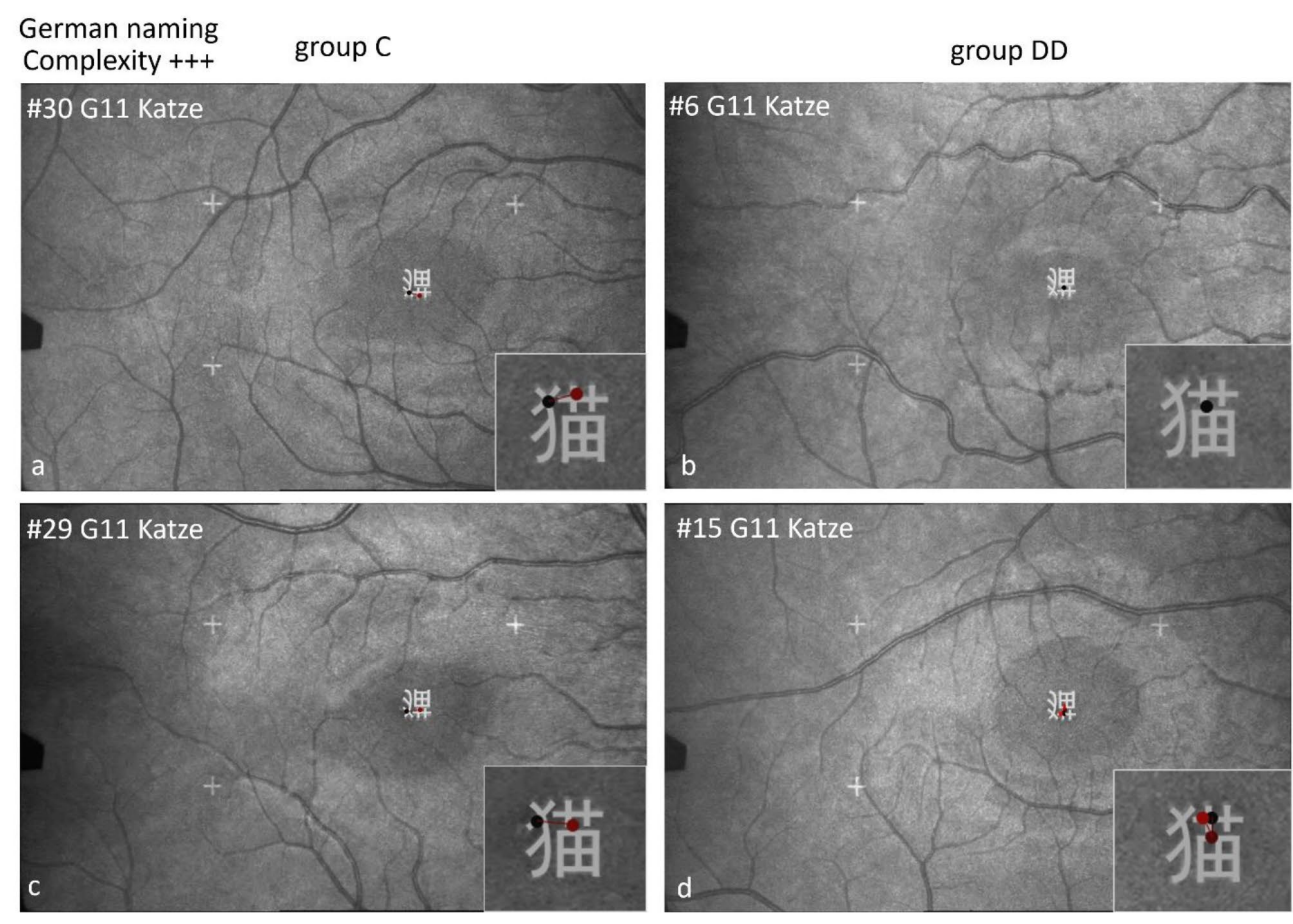
Individual scan-paths when naming Chinese characters of high visual complexity in German. The character “cat” (Katze) contains a special feature: The sub-grapheme “wild animal”. The 2 children of group C look first at the sub-grapheme and then to the main part of the character for specification. The 2 children of group DD fixate first in the center of the character.

Figures 9, 10 and 11 show examples of scan paths used to name Chinese characters with increasing visual complexity in German, on the left for group C, on the right for group DD. It is evident that the characters were fixated either by only 1 fixation (Figs. 9 and 10 upper row), or by 2 or more fixations (Figs. 9, 10 and 11, lower row), which was similar in children of both groups. Visual complexity did not play a major role.

Figure 11 shows scan paths while naming Chinese characters in German with high visual complexity. In both groups, an *individual* character can be examined with only 1 or 2 fixations (upper row) or with more fixations (lower row).

*Location of first fixation*: Children of group C located their first fixation more frequently on the left part of the characters, but those of group DD on the center (for details see Table 6 below). This behavior becomes especially evident in characters with a sub-grapheme. The character “cat” (here to be named in German) has a special feature: A left-right structure with the sub-grapheme on the left (犭”wild animal“) and the specifying part on the right. The 2 children of group C looked first at the sub-grapheme 犭and then at the main part of the character for differentiation. The 2 children of group DD first fixated the center of the character. Both can be done with 1–2 (upper row) or with more fixations (lower row).

### Naming Chinese characters in Chinese

**Fig. 12:**
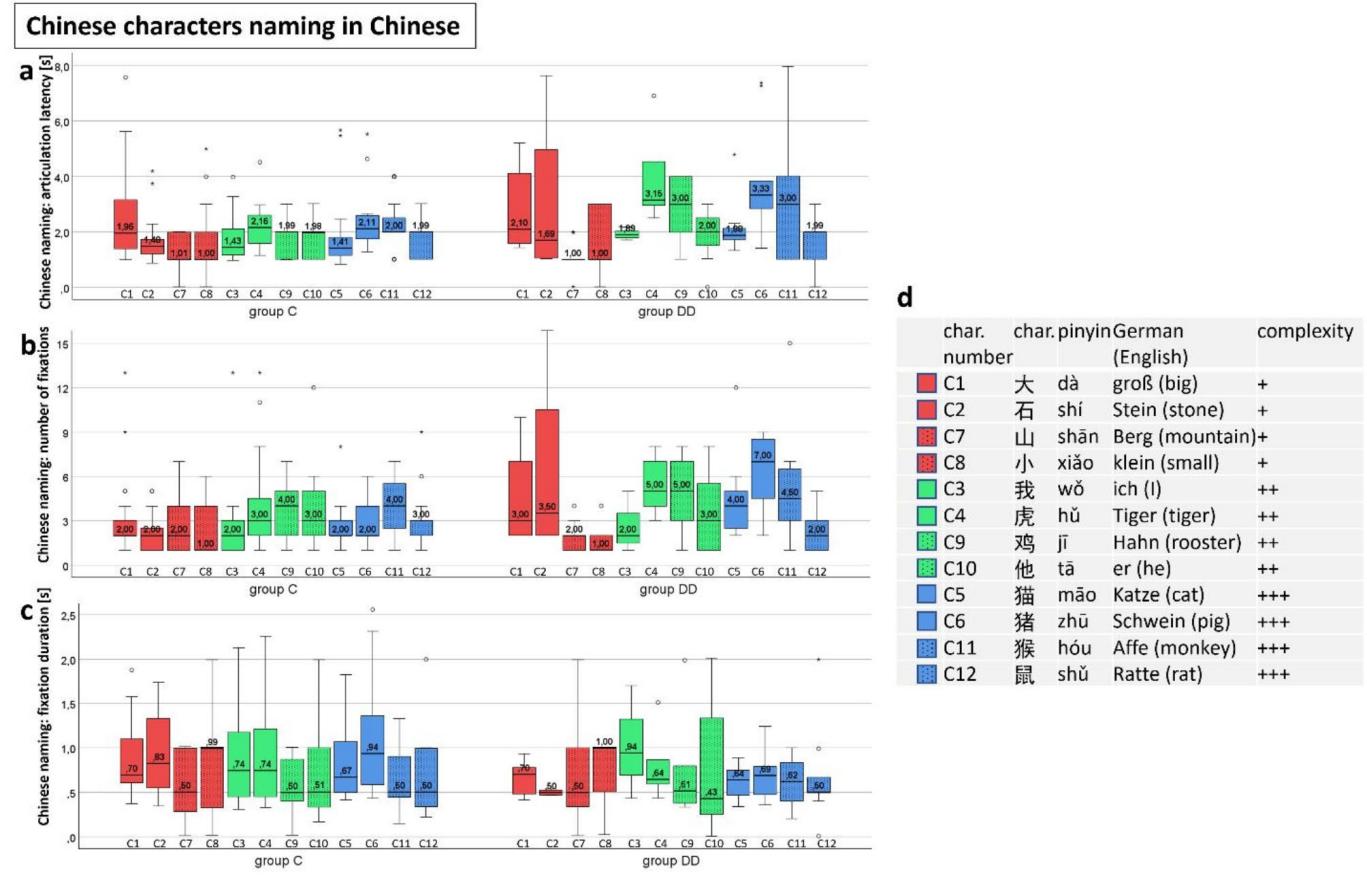
Naming Chinese characters in Chinese. (**a**) Articulation latency, (**b**) number of fixations, (**c**) fixation duration, (**d**) Chinese characters with increasing visual complexity. For most of the stimuli, all three variables did not differ significantly between the two groups, except characters C4-C6 regarding articulation latency and C5-C6 regarding the number of fixations. See also Table 5.

**The articulation latency** (Fig. 12a) showed higher values for several characters in group DD: The group differences were significant for the characters **虎 tiger (C4) and 猫 cat (C5) and 猪 pig (C6) -** for statistics see Table 5. Group C showed a moderate, significant correlation between articulation latency and visual complexity (see paragraph “visual complexity” below).

**The number of fixations** (Fig. 12b) was significantly higher in group DD for the characters **猫 cat (C5) and 猪 pig (C6)** - see Table 5. Both groups showed a moderate, significant correlation between number of fixations and visual complexity (see paragraph “visual complexity” below).

**The differences of the fixation durations** between the groups (Fig. 12c) were not significant and there was no influence of visual complexity.

The main variables of all three “difficult” characters again showed the tendency of group DD to increase the number of fixations, and that of group C to prolong fixation duration (see Table 5) or articulation latency.

**Table 5:**
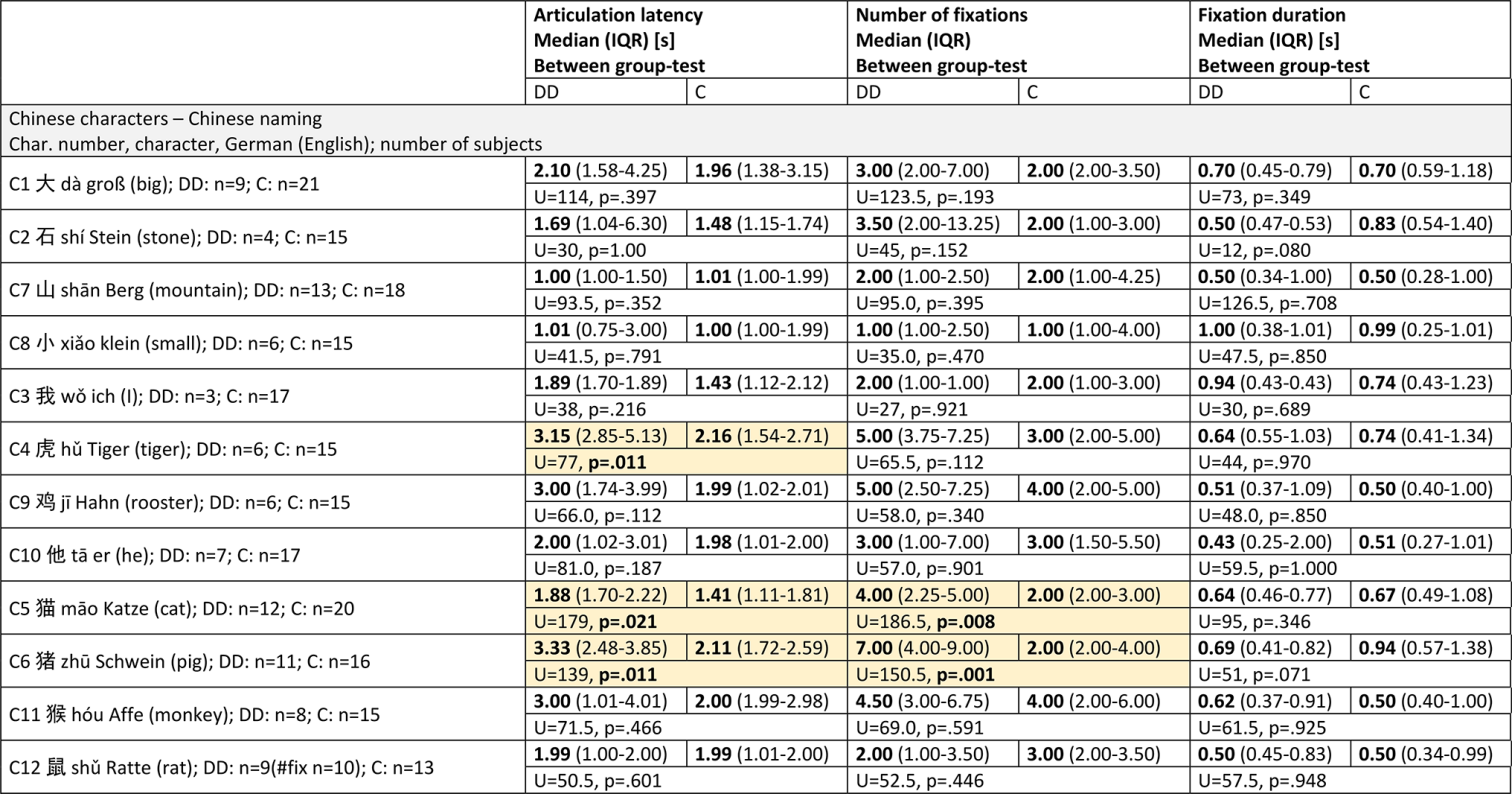
Chinese characters, Chinese naming.

DD= children with dyslexia, C= control group, n= number of subjects, IQR= interquartile range, U= Mann-Whitney U-test statistics U-value, p= probability value, s= seconds, between-group-test= Mann-Whitney U-test. The characters were presented with increasing visual complexity, C1-C6 with the Infrared Eye Tracker, C7-C12 with the SLO. The yellow fields highlight the significantly different results.

#### Scan paths during EM recording by SLO while naming Chinese characters in Chinese

**Fig. 13:**
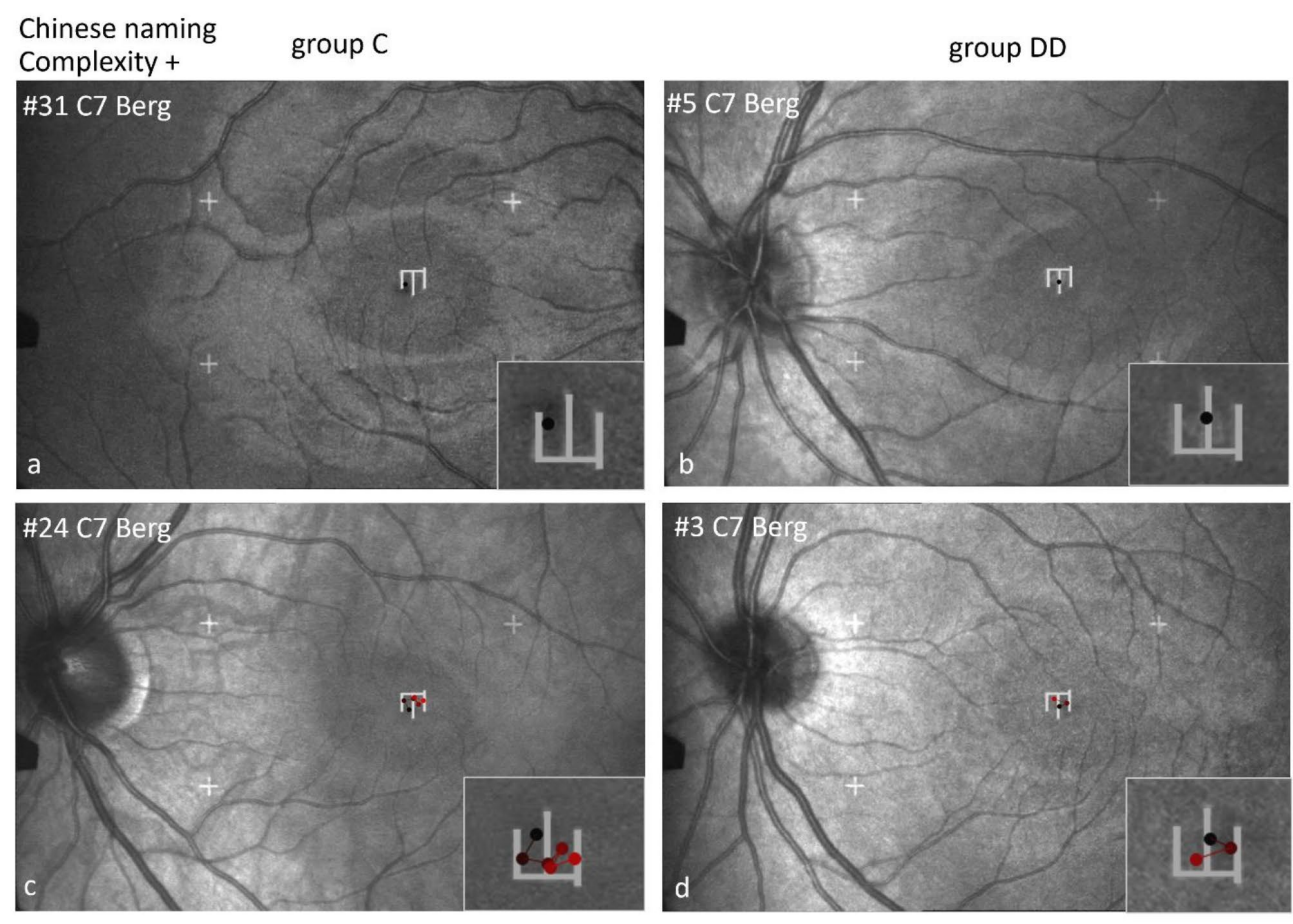
Individual scan-paths when naming Chinese characters in Chinese of low visual complexity. Two examples from each group are shown for the same character, in the upper row with only 1 fixation, in the lower row with 4–5 fixations with no difference between the groups.

**Fig. 14:**
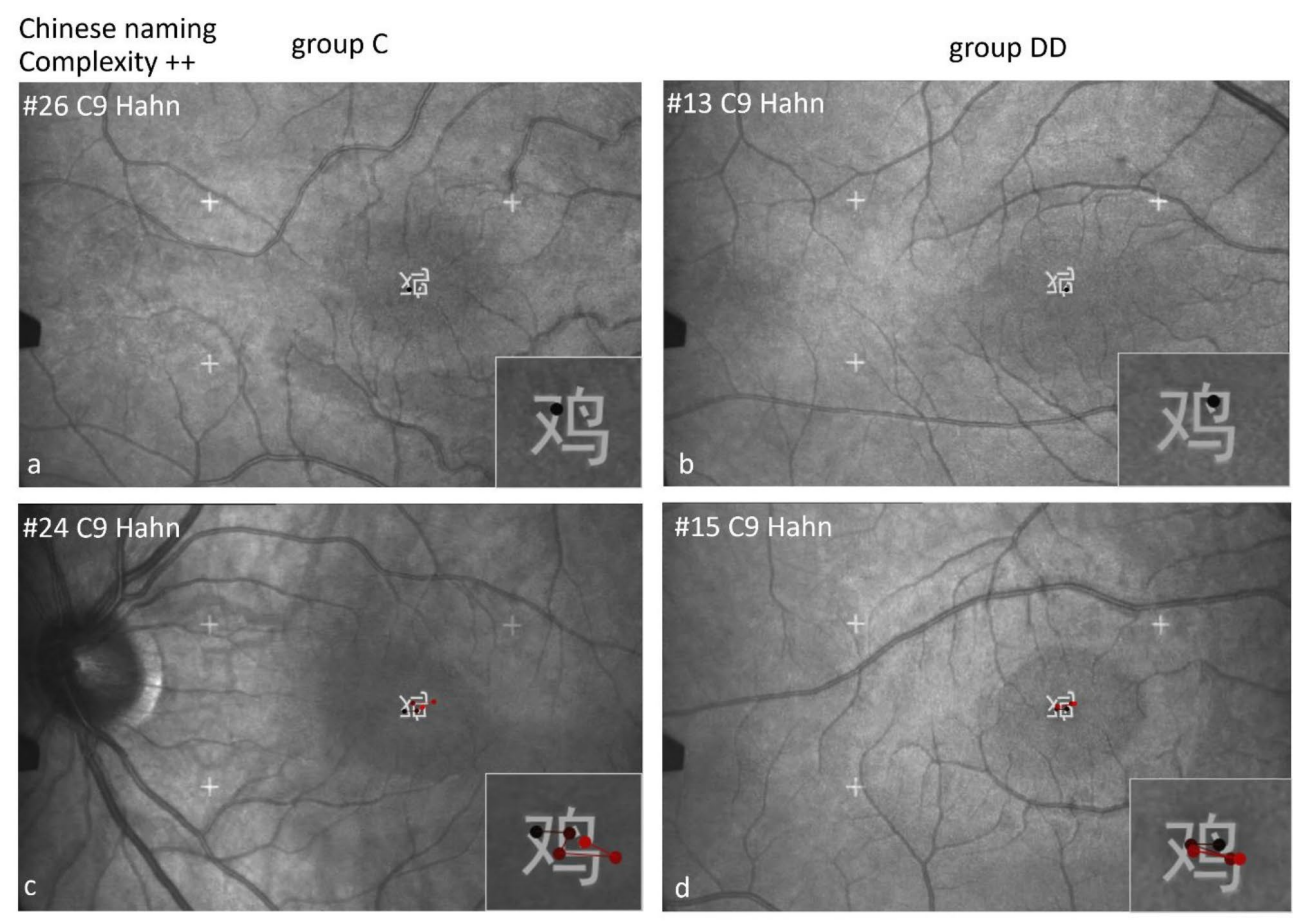
Individual scan-paths when naming Chinese characters in Chinese of medium visual complexity. Two examples from each group are shown per character, in the upper row with only 1 fixation, in the lower row with 4–5 fixations with no difference between the groups.

**Fig. 15:**
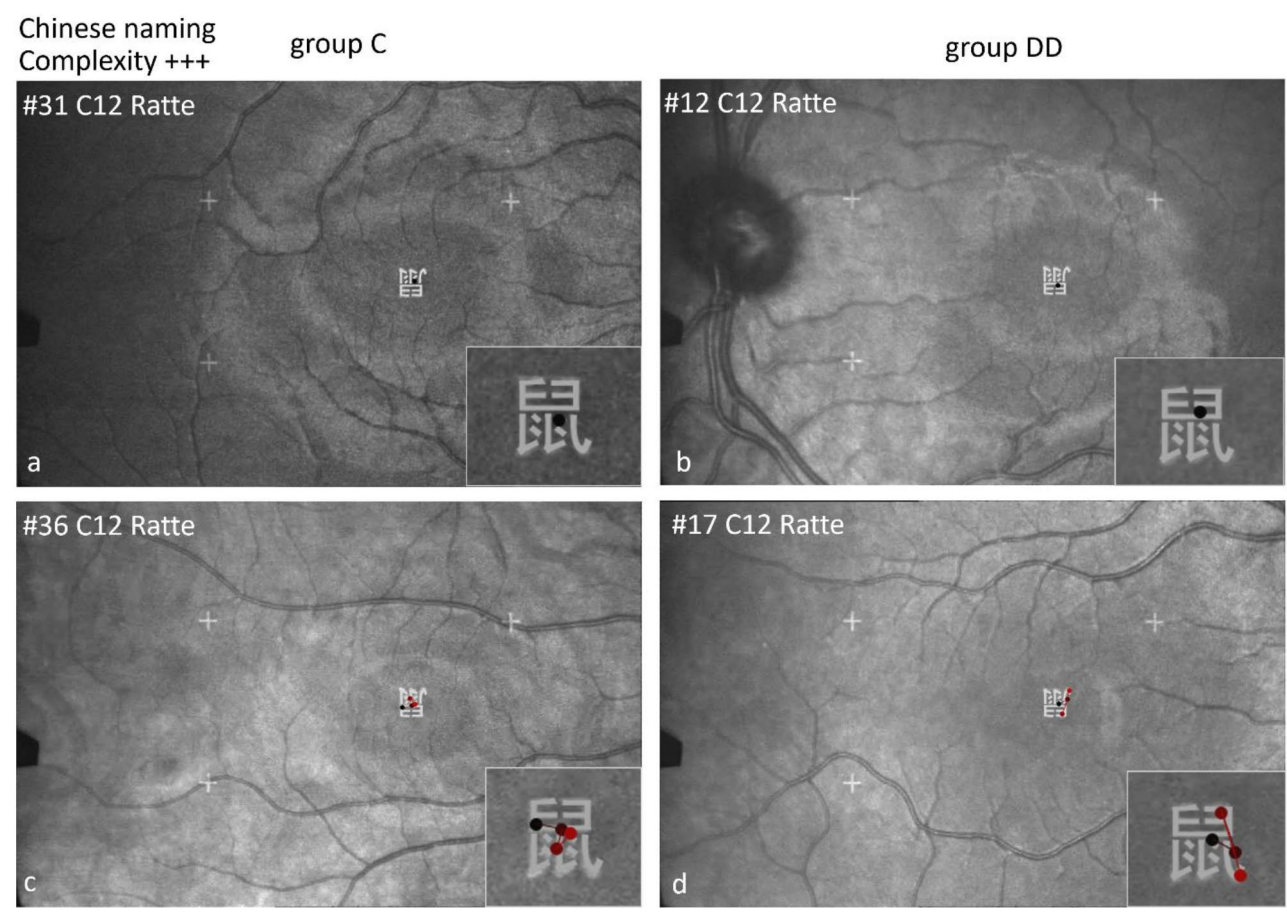
Individual scan paths when naming Chinese characters in Chinese of high visual complexity. The number of fixations was not different between the groups: The upper row shows examples with only 1 fixation, the lower row some with 4–5 fixations.

**Fig. 16:**
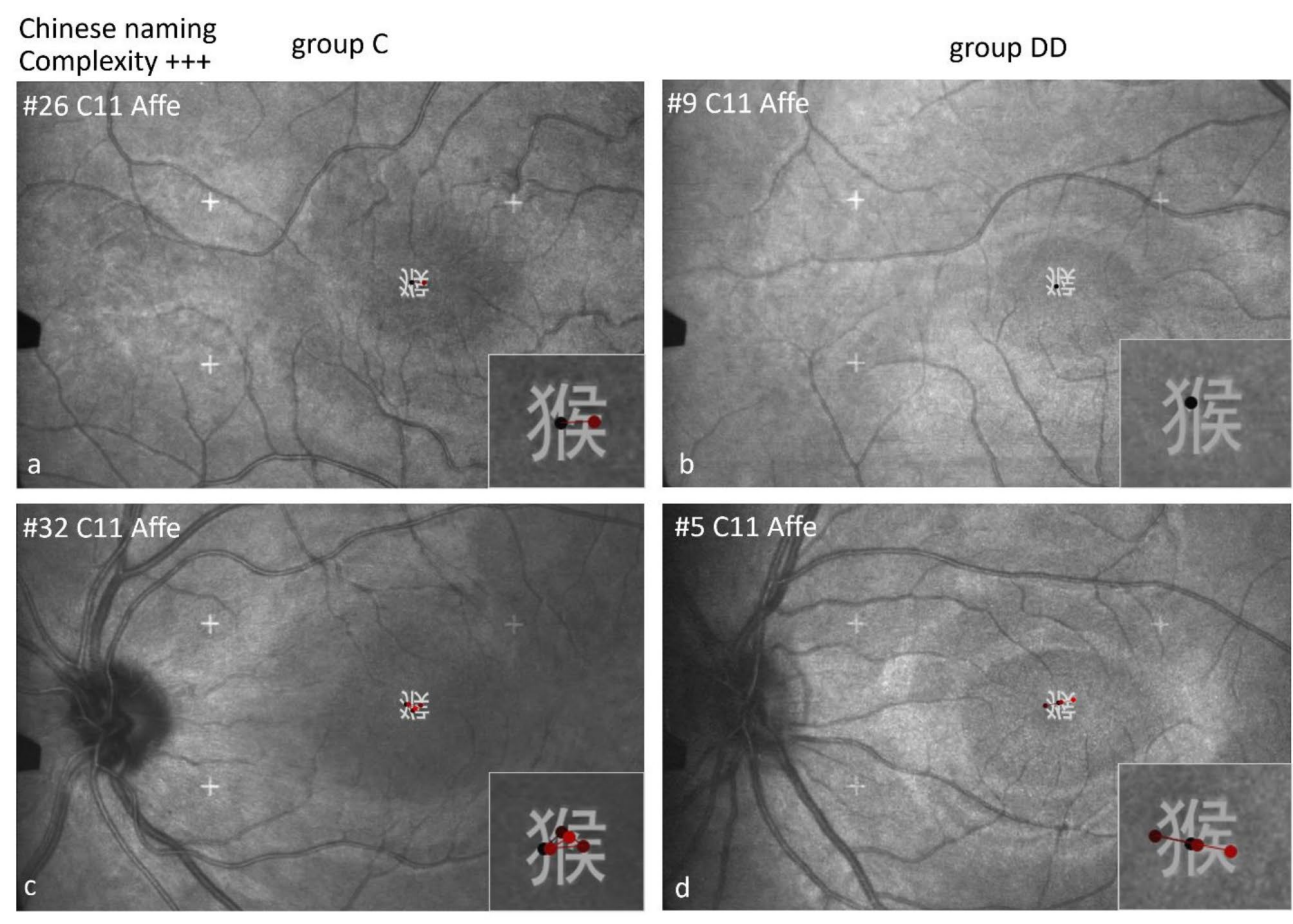
Individual scan-paths when naming Chinese characters in Chinese of high visual complexity and a special structure. The figure shows 1 or 2 fixations in the upper row, with 4–5 fixations in the lower row without a difference between the groups. The character 猴 (monkey) has a left-center-right structure, i.e. on the left the sub-grapheme 犭, in the center a vertical structure and then a right part. The figure shows examples of children of both groups with the first fixation on the central vertical structure (**a**, **b**, **d**) and a child of group C with the first fixation on the sub-grapheme (**c**).

Figures 13, 14, 15 and 16 show examples of the scan paths while naming Chinese characters of increasing visual complexity in Chinese for children of group C (left), and of group DD (right). Regarding the *number of fixations*, children of both groups used only one fixation (Figs. 13, 14 and 15, upper row), while others used more fixations (lower row).

Also in the Chinese naming task, the children of group C located their *first fixation* more frequently on the left part of the characters, while those of group DD on the center (see Table 6). The difference between the groups for both naming tasks together was significant (*p* = .001).

The pictographic character 鼠 (rat, C12) can be named using only 1 fixation (Fig. 15a,b, upper row), in others only after 4 fixations (Fig. 15c,d, lower row). This shows again that visual complexity did not play a major role.

The character 猴 (Affe, monkey, C11) has the same sub-grapheme as cat and pig (Fig. 16). However, it has a left-center-right structure with an additional vertical structure beneath the sub-grapheme, both of which make the task more difficult. Children of both groups preferred to fixate first the central vertical structure, more often in group DD (for details see Table 6). The differences between the groups were not significant. Figure 16 shows examples of children of both groups with the first fixation on the central vertical structure (a, b, d) and of a child of group C with the first fixation on the sub-grapheme (Fig. 16c).

### Summary of the results of the EM variables

We found only few significant differences between the groups: For German naming, in group DD articulation latency was longer in 猫 (G11, cat), number of fixations was higher in 龙 (G3, dragon), whereas fixation duration was shorter in 属 (G5, to belong to). For Chinese naming, in group DD articulation latency was longer in 虎 (C4, tiger), 猫 (C5, cat) and 猪 (C6, pig). and number of fixations was higher in 猫 (C5, cat) and 猪 (C6, pig) see Tables 4 and 5.

#### Location of first fixation

For naming characters in German and Chinese, the majority of the children of group C located their first fixation on the left part of the characters, but those of group DD on the center (for details see Table 6). The difference between the groups for both naming tasks together was significant (*p* = .001). This behavior becomes especially evident in characters with a sub-grapheme, such as 猫 (cat, Fig. 11, German naming) and 猴 (monkey, Fig. 16, Chinese naming).

**Table 6:** First fixation.

	Group DD				Group C			
	Left	Center	Ambiguous	Sum	Left	Center	Ambiguous	Sum
Number of children	42	67	2	111	113	81	11	205
Percentage of children	37.8	60.4	1.8	100	55.1	39.5	5.4	100

„First Fixation” shows the number and percentage of the children, who located their first fixation either the left or center of a Chinese character. “Ambiguous” were cases where it was not possible to categorize the location of first fixation. DD= children with dyslexia, C= control group. A chi-square test showed that there was a significant difference between groups and the preference for the first fixation on a Chinese character being either left, center, or ambiguous, χ2(2, *n*=316) = 13.292, *p*=.001, Cramér’s phi= 0.205 (medium effect).

### Correct answers while naming Chinese characters in German and Chinese

**Fig. 17:**
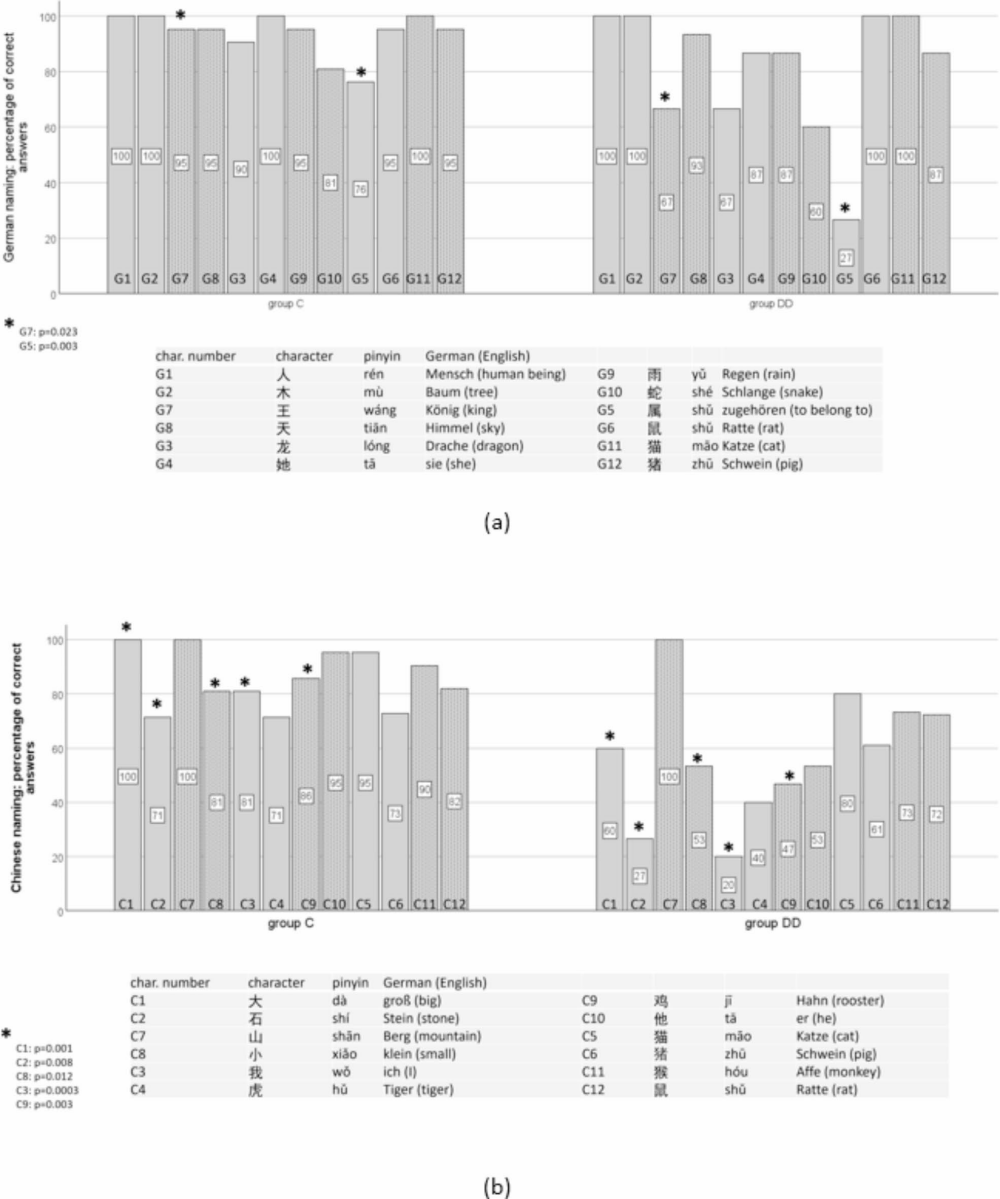
Percentage of correct answers when naming Chinese characters in German (**a**) and in Chinese (**b**). The characters are displayed with increasing visual complexity. Children of group DD make significantly more mistakes for characters G5 and G7 during German naming (**a**), and C1-3 and C8-9 during Chinese naming (**b**) than those of group C. For details see text.

Figure 17a and b show the percentage of correct answers when naming Chinese characters in German (a) and in Chinese (b). In group DD, significantly more characters were named incorrectly or not at all. Therefore, only the correct answers could be analyzed as EM variables.

Of the characters to be named in German (Fig. 17a), 属 (G5, to belong to) and 王 (G7, king) were incorrectly or not named by members of both groups, but significantly more often in group DD. In the Chinese naming task, group DD showed significantly fewer correct responses for characters 大 (C1, big), 石 (C2, stone), 我 (C3, I), 小 (C8, small) and 鸡 (C9, cock) (Fig. 17b).

### The influence of visual complexity

For German naming, the number of pixels correlated moderately with the number of fixations in group DD (Spearman rho = 0.613, *p* = .034) – see Table 7. For Chinese naming, the number of pixels correlated moderately with articulation latency in group C (rho = 0.627, *p* = .029) and with the number of fixations in group DD (rho = 0.597, *p* = .040).

**Table 7:** Visual complexity correlations.

Number of pixels	German naming Articulation latency	German naming Number of fixations	German naming Fixation duration	German naming Correct answers
Group DD	Rho= 0.242, *p*= .449	**Rho= 0.613**, *p*= .034	Rho= -0.477, *p*= .117	*r*= -.335, *p*= .287
Group C	Rho= 0.106, *p*= .743	Rho= 0.506, *p*= .093	Rho= 0.183, *p*= .568	Rho= -0.351, *p*= .263

	Chinese naming Articulation latency	Chinese naming Number of fixations	Chinese naming Fixation duration	Chinese naming Correct answers
Group DD	Rho = 0.567, *p*= .054	**Rho = 0.597**, *p*= .040	Rho= -0.063, *p*= .845	*r*= .059, *p*= .856
Group C	**Rho= 0.627**, *p*= .029	Rho= 0.494, *p*= .103	Rho= 0.004, *p*= .991	Rho= -0.197, *p*= .539

Correlations between number of pixels and EM variables in German and Chinese naming tasks. Number of characters *n*=12, DD= children with dyslexia, C= control group, n= number of subjects, Rho= Spearman’s rank correlation coefficient, r= Pearson correlation coefficient, p= probability value. The same value (number of pixels for Chinese character stimuli) is being correlated with each of the values across top row, but in 2 different settings: German naming of stimuli and Chinese naming of stimuli. Group DD shows a positive, significant correlation of the number of pixels with the number of fixations in both naming tasks, group C with articulation latency only during Chinese naming.

### The influence of the phonological deficit in group DD

We looked for correlations between indicators of the phonological deficit and the results of the EM variables and the error rate to demonstrate the effect of the phonological deficit of the children in group DD on their poorer performance in naming individual characters. This was done by using three approaches:

1. Correlation of the EM variables during naming alphabetic words - as a measure of the severity of DD - versus the EM variables during German and Chinese naming: We found no correlation in either group or task (Table 8).
2. Correlation of the number of fixations during naming alphabetic words versus the number of correct answers during German and Chinese naming: There was no correlation in either group or task (Table 9).
3. Correlation of reading speed at baseline versus the number of correct answers: Again, no correlation in either group or task (Table 9).

**Table 8:** Phonological correlations: *EM variables while reading alphabetic words as a measure of the phonological deficit versus EM variables while naming Chinese characters: no correlations*,* no significant values*.

	Articulation latency		Number of fixations		Fixation duration	
	Group DD	Group C	Group DD	Group C	Group DD	Group C
German naming						
Articulation latency	Rho=-0.118, *p*= .700, *n*=13	Rho= 0.116, *p*= .627, *n*=20	Rho=0.108, *p*= .726, *n*=13	Rho= -0.078, *p*= .745, *n*=20	Rho= -0.278, *p*= .358, *n*=13	Rho= 0.182, *p*= .442, *n*=20
Number of fixations	Rho= -0.188, *p*= .539, *n*=13	Rho= 0.069, *p*= .774, *n*=20	Rho= -0.059, *p*= .865, *n*=13	Rho= 0.224, *p*= .342, *n*=20	Rho= -0.075, *p*= .808, *n*=13	Rho= -0.258, *p*= .273, *n*=20
Fixation duration	Rho=0.080, *p*= .796, *n*=13	Rho= 0.199, *p*= .399, *n*=20	Rho= 0.277, *p*= .360, *n*=13	*r*= -.199, *p*= .401, *n*=20	*r*= -.069, *p*= .823, *n*=13	*r*= .336, *p*= .148, *n*=20
Chinese naming						
Articulation latency	Rho= -0.358, *p*= .310, *n*=10	Rho= -0.148, *p*= .559, *n*=18	Rho= 0.000, *p*= 1.00, *n*=10	Rho= 0.023, *p*= .926, *n*=18	Rho= -0.273, *p*= .446, *n*=10	Rho= -0.018, *p*= .945, *n*=18
Number of fixations	Rho= -0.148, *p*= .684, *n*=10	Rho= -0.030, *p*= .907, *n*=18	Rho= 0.417, *p*= .231, *n*=10	Rho= 0.193, *p*= .442, *n*=18	Rho= -0.283, *p*= .428, *n*=10	Rho= -0.317, *p*= .200, *n*=18
Fixation duration	Rho= 0.200, *p*= .580, *n*=10	Rho= -0.046, *p*= .855, *n*=18	Rho= -0.480, *p*= .160, *n*=10	Rho= -0.188, *p*= .642, *n*=18	Rho= 0.467, *p*= .174, *n*=10	Rho= 0.095, *p*= .708, *n*=18

DD= children with dyslexia, C= control group, n= number of subjects, Rho= Spearman’s rank correlation coefficient, r= Pearson correlation coefficient, p= probability value.

**Table 9:** Phonological correlations: reading speed at baseline versus correct answers: *no correlation in either group or task*

	Group DD	Group C
Correct answers German naming		
Alphabetic words number of fixations	Rho= 0.167, *p*= .553, *n*=15	Rho= 0.038, *p*= .872, *n*=21
ZLT reading speed	Rho= -0.059, *p*= .833, *n*=15	Rho= -0.014, *p*= .951, *n*=21
Correct answers Chinese naming		
Alphabetic words number of fixations	Rho= -0.071, *p*= .800, *n*=15	Rho= 0.151, *p*= .513, *n*=21
ZLT reading speed	*r*= .069, *p*= .806, *n*=15	Rho= -0.053, *p*= .819, *n*=21

DD= children with dyslexia, C= control group, n= number of subjects, Rho= Spearman’s rank correlation coefficient, r= Pearson correlation coefficient, p= probability value, ZLT= Zürcher Reading Test.

## Discussion

To summarize the results of the *Chinese character naming tasks* regarding our research questions in the Introduction, we found:

*For both groups*:

Conspicuous features of the Chinese characters influence EM variables – for details see below.

Furthermore, EM patterns and scanning strategies provided evidence that Chinese characters are processed mainly holistically. Some children could recognize a character with just 1 fixation, while others needed 2 or more.

*Differences between the groups*:

EM variables showed only a few significant differences in the Chinese character naming tasks. However, the children in group DD had a significantly higher error rate.

Scanning behavior showed a significant difference between the groups regarding the location of the first fixation: Children with DD were more likely to fixate first the center of the character, those of group C the left part (Table 6).

*A general strategy for all kinds of stimuli* was observed, when a stimulus appeared to be “difficult”: The children of group DD displayed a general response pattern in all tasks by naming specific stimuli with a higher number of fixations, whereas the control group (C) tended to extend the fixation duration or articulation latency.

### Findings and discussion relevant to our hypotheses

#### Hypothesis 1: Children with DD perform worse than those without DD when naming alphabetic words in regard to their EM variables

This hypothesis has been confirmed: We focused on each single word and found highly significantly more fixations and longer articulation latencies in members of group DD, which increased with growing phonological difficulty.

This finding confirmed results reported in the literature^23,30,45^, as well as our own previous studies on alphabetic reading^20,35,36,46^. The scan paths seen in the SLO images (Figs. 3, 4 and 5) illustrate the difficulties encountered by the children with DD. This difficulty in grapheme-phoneme conversion with many fixations within one word, is typical for reading words in a regular orthography, such as German^6–8,30,45^. It indicates that children with DD in an alphabetic language process words in smaller alphabetic units, already in short and frequent words, but much more pronouncedly in long and rare words. (Figures 2, 3, 4 and 5; Table 2).

#### Hypothesis 2: Children with DD perform similarly to the group without DD in the logographic naming tasks in regard to their EM variables

This hypothesis has been confirmed for pictures as well as for Chinese characters: On average there were no significant differences between the groups, for individual stimuli rarely a higher number of fixations occurred in group DD.

*Pictures*: In agreement with our previous studies^20,27^ we found no major differences between the groups, which supports the hypothesis that pictures are processed primarily via the visuo-spatial pathway to access their meaning. This result also confirmed that these children did not have a visual deficit, which was also mentioned in other studies^22,47,48^. Only the picture “banana” was named with significantly more fixations in group DD. This was compensated by a significantly shorter fixation duration, which resulted in a normal articulation latency (Figs. 6 and 7 also Table 3). The finding of unimpaired “confrontational” picture naming in our previous studies^20,27^ raised the question, whether this task might serve as a predictor for good capabilities to learn Chinese for children with DD who were educated in an alphabetic writing system. Both tasks are processed primarily by the visuo-spatial pathway with direct access to the meaning. However, it should be considered that only few logographic stimuli are purely pictographic. It has been reported that in the process of learning Chinese at an early age (below second grade), visual skills seem to predict successful reading, which was interpreted as a contribution by a logographic/visual phase^3,49^. Our recent publication^29^ reported a significant positive correlation of the number of fixations during picture naming with the number of fixations during naming Chinese characters in Chinese for group DD, but not for group C. This can be interpreted as a predictor of good capabilities to learn a logographic script, but the number of pictures tested was too small to allow a conclusion. This is why future studies should examine whether a pre-test with more pictures might show that the children with DD have good chances to learn Chinese. It could benefit children with DD to learn a second language successfully and with enjoyment, after they have had many negative learning experiences with an alphabetic language before. The subjective reports of the children in our recent report^29^ were positive, because most children said that they had fun learning the Chinese characters during the lessons.

*Chinese characters*: We found only few significant differences between the groups regarding the EM variables, which showed that the children with DD had much less problems with the Chinese naming task than with the alphabetic word reading. Even though there is considerable evidence that phonology plays a minor role in logographic writing systems^4,5^, producing a verbal answer is still dependent on recall or assembly of a phonological representation. The good performance in the Chinese character naming tasks may be influenced by the lack of stress on recall of orthography to phonology connections in these two tasks, which is where the phonological deficit of DD children typically manifests most significantly.

Other EM studies have examined reading compound words or sentences, e.g^5,50–52^. They examined mostly persons who were familiar and experienced with reading Chinese characters. These studies are not directly comparable with the current study, because we presented only one-character words, and we examined children who are absolute beginners. This allowed us to observe their spontaneous approach to a new writing system.

#### Hypothesis 3: Children with DD show higher error rates than those without DD when naming Chinese characters

This hypothesis has been confirmed: The error rate was significantly higher in children of group DD, more pronounced in the Chinese than in the German naming task.

Of the characters to be named in German (Fig. 17a), G5 (to belong to) was associated with incorrect answers in both groups, but significantly more often in group DD. Indeed, the meaning of this character is rather abstract, it is one of only two verbs, and it was introduced in a later period of the lessons, so that it was less familiar. The character 王 (king, G7) was also correctly named significantly less often in group DD. The reasons could be the similarity with the characters 大 dà (big, C1), 天 tiān (sky, G8) and the fact that this character was taught in the very last lesson and was therefore less familiar than others.

Most of the children of group DD named the Chinese character 我 wǒ (C3, I) incorrectly or not at all. This may either be due to confusing the left part of the character, which might be taken for the sub-grapheme 犭“wild animal”, or to the lack of a clear center, which would be the preferred first location to fixate in presence of a sub-grapheme. Furthermore, the frequent changes in stroke direction may be difficult during writing the character. Stroke order has been reported to be essential for character writing^53^. This is relevant here, because the lessons in our study also included character writing. Learning the Chinese characters through calligraphy by training manual memory makes an important contribution to memorizing them^16,17^.

We observed a special behavior in the children with DD during naming some Chinese characters: Either they named the character spontaneously and quickly (sometimes even faster than those in group C), or they promptly said “I don´t know”. The children in group C, on the other hand, made a bigger effort and took more time to name the character. The answer “I don’t know” was given by 24% of the children of group DD, but only by 7% of group C. An incorrect answer was given by 15% of group DD, but only by 5% in group C^29^. Based on this behavior, we assume that the lower percentage of correct answers in group DD might be influenced by lowered self-esteem, impaired memorization, and lack of motivation based on negative language learning experiences, rather than by a phonological deficit. Furthermore, we do not think that an essential difference between the amount of instruction during the lessons or use of the app contributed to the results. Our finding that children of group DD more often showed restless behavior during EM recording, which could cause artefacts, might indicate reduced attention and concentration, even though diagnosed ADHD was an exclusion criterion in our recruiting for this study.

In future studies, it would be interesting to find out whether a longer learning period (here 8 × 3 h) can improve the performance of children with DD by giving them more time, or whether this might increase the difference between the groups.

Discussing the influence of the phonological deficit in alphabetic languages on learning Chinese has caused controversy. Several publications reported that children with an education in an alphabetic script as a first language (L1), who later learned Chinese, also had problems that were attributed to their phonological deficit, at least partly^18,54^. Other authors have emphasized the importance of morphological awareness^10,19,55^, visuo-spatial cognitive processing^56,57^ and copying skills^4,16,17^.

It could be suspected that the phonological deficit of our children with DD was the cause for the higher error rate in the current study. Therefore, we examined potential correlations of the phonological deficit of the children of group DD with their naming performance. We used the number of fixations during naming alphabetic words as a measure of the phonological deficit, because it is markedly increased in this task and rises with growing phonological difficulty of the words. We found neither a correlation with the number of fixations during the German and Chinese naming tasks, nor with the number of correct answers. Although we are aware that the severity of DD is not only based on reading speed, we applied reading speed measured by Zürcher Reading test as an additional measure of phonological performance. In a regular orthography such as German, the children with DD primarily read more slowly and with few mistakes, in contrast to children reading in an irregular orthography, such as English, who make more mistakes^6–8^. For both measures, we did not find a correlation with German and Chinese naming performance. Therefore, considering the naming tasks used in the current study, the phonological deficit of the children of group DD does not seem to play an essential role in causing their lower performance. This could be different in tasks that require pronouncing unfamiliar sounds or tones.

#### Hypothesis 4: Pictures and Chinese characters are processed as a whole by the visuo-spatial pathway

This hypothesis has been confirmed: Children of both groups processed the pictures and the Chinese characters with one or only few fixations.

The scan paths showed in b*oth groups similar behavior* regarding the *number of fixations* while naming Chinese characters in German and in Chinese: Some children fixated a character only once (Figs. 9, 10, 11, 13, 14, 15 and 16, upper row), others two or more times (lower row).

Characters of medium or high visual complexity (e.g. cat, rain, monkey) are examined with fewer fixations by some children of group DD than by those of group C (e.g. Figures 10d, 11b, 14d and 16b,d). This indicates the primarily visual approach by children of group DD – who see this character as a “symbol”, not as a word.

The groups showed a significantly *different behavior* regarding the *location of the first fixation* (Table 6): The children of group C were more likely to fixate first on the left part of the character and then made the next saccade to the right, as they usually do in alphabetic reading. The children of group DD were more likely to fixate first in the center of the character, which indicates mainly visuo-spatial, holistic processing. This behavior is especially evident during naming characters with a sub-grapheme.

*Different behavior for characters including the sub-grapheme* 犭 *“wild animal”.* The majority of the children of group C fixated first the sub-grapheme and secondly the specifying center (right part) of the characters cat and pig, which is a similar linear analytic decoding behavior from left to right- as in scanning alphabetic words. In contrast, children of group DD were more likely to fixate the center of the character directly, which indicates a holistic approach. Children of group C were able to use a proven strategy of linear analytic scanning, while children of group DD mainly used a holistic approach relying on visual processing.

Whereas the characters cat and pig contain two parts in a left-right structure, the character **monkey** contains 3 parts in a left-center-right structure (Fig. 16). The second part has an additional vertical stroke structure just beneath the sub-grapheme **犭**, which makes it more difficult to recognize the sub-grapheme. Here, the children of both groups preferred to first look at the central structure, more often in group DD.

Visual complexity was only moderately correlated with the EM variables and therefore did not play a major role for the performance in the Chinese character naming tasks. It is conceivable that the analysis of a highly complex character requires more effort for members of both groups, only the strategy was different: Group C increased the articulation latency during Chinese naming, group DD the number of fixations in both naming tasks (Table 7). However, visual complexity was not correlated with the error rate.

The minor role of visual complexity indicates that the Chinese characters can be processed by an unimpaired visuo-spatial pathway. Otherwise, there could have been interference by crowding, a visual deficit reported in several studies^24,58^. We did not find enhanced crowding in this cohort in our previous report^29^, and it has been hypothesized that it might occur only in subgroups^20,25,26^. The unimpaired visual capabilities might ease the use of holistic processing of the characters in the children with DD.

#### Hypothesis 5: Naming performance does not depend on the visual complexity of Chinese characters

This hypothesis must be partially rejected:

We found a moderate influence of visual complexity on EM performance in both groups, but no correlation with the error rate.

### Possible reasons for difficulties with naming individual Chinese characters

#### Characters of medium visual complexity

The character 龙 lóng (dragon, G3) was named in German with significantly more fixations in group DD. This character has only 5 strokes, but low structure and no conspicuous features to aid recognition. As in the other tasks, it was a general behavior pattern that children of group DD preferred to make more fixations if they found a task more difficult. However, this is compensated by a shorter fixation duration than that of group C, which caused the articulation latency not to differ significantly between the groups (see Table 4).

If the character 虎 hǔ (tiger, C4) is to be named in Chinese, it does not provide conspicuous features for memorization. Both groups responded with long articulation latencies (see Table 5), but they were significantly longer for group DD. In this case, the shorter fixation duration could not fully compensate for the increased number of fixations. However, only 6 children of group DD named it correctly.

#### Characters of high visual complexity

Four of the visually very complex characters, namely **猫** (cat), **猪** (pig), **猴** (monkey) and **狗** (dog – learned in class, but not tested during EM recording) contain the same conspicuous feature, which can lead to confusion: the sub-grapheme **犭**on the left, which means “wild animal”. The challenges for the learners were therefore: to recognize this element, to identify the right part of the character, and to differentiate this part from the three other characters. In the lessons, memory aids were provided.

**The character 猫 māo (cat**,** G11 and C5)** was to be named in German and in Chinese. While naming it in German (G11), both groups gave 100% correct answers, which indicates that recognizing the character’s meaning was no problem, but group DD showed a significantly longer articulation latency. During Chinese naming (C5), incorrect answers were given by one child of group C and by two children of group DD. The cat was an important “Figure” in the story of the lessons. The sound of the Chinese character 猫 māo is very easy to memorize and to pronounce (considering the German sound for meowing “miau”). Therefore, it is unlikely that the lower performance in children of group DD for this character is caused by a phonological problem. The longer articulation latency and the higher number of fixations in group DD during Chinese naming (C5) can therefore be explained by the difficulty to differentiate the character from the others with the same sub-grapheme 犭wild animal”.

**The character 猪 zhū (pig)** was also to be named in German and in Chinese. Its EM variables did not differ during German naming (G12), but produced longer articulation times and more fixations during Chinese naming (C6) in group DD. The percentage of correct answers was lower during Chinese naming than during German naming in both groups, but the difference between the groups was not significant. The Chinese pronunciation of this character includes an initial sound that is rather difficult to produce for beginners. Thus, in addition to discrimination of the sub-grapheme, the phonological deficit may play a role for the reduced performance in group DD.

**The completely different character 属 shǔ (to belong to**,** G5)** presented difficulties for both groups, because it was unfamiliar, it was one of only 2 verbs that were taught (beside the connector “to be”), and it occurred rather late in the lessons (see Table A3 in the Appendix). Note that its naming is associated with a significantly shorter fixation duration in group DD. However, only 4 children with DD gave the correct answer - the lowest percentage of all characters (see below).

### The two writing systems require different skills

**Reading and naming alphabetic scripts** require phonological decoding and are mainly based on the sounds of characters and syllables. In many alphabetic orthographies, phonological awareness is essential for grapheme-phoneme conversion^6–8^, and phonological working memory (the phonological loop) is important for storing and processing language information simultaneously^9^.

The children of group DD revealed their phonological deficit during naming alphabetic words primarily by increasing the number of fixations with increasing phonological difficulty of the words, which also led to a prolonged articulation latency. This confirms the results of our previous studies^20,35,36,46^ and publications by other authors^6,7,45^.

**Reading logographic scripts** requires primarily visual skills: Visual perception, visual memory, and visuo-spatial capabilities are important at the beginning of learning Chinese^49,59^. Furthermore, morphological awareness is an essential requirement^4,5,10–16^ for analyzing the morphological structure of a character. A morpheme is the smallest meaningful or consistently occurring unit in a language. In addition, copying Chinese characters was found to be important for Chinese children who learn to read, because it points to differences and similarities between characters^16,17^. The process of learning Chinese characters concentrates on their meaning rather than on their sounds^16^.

Chinese children with dyslexia have been reported to show deficits mainly in morphological awareness, visuo-orthographic knowledge as well as impaired copying^4,5,10–16^. Children with DD, who are educated in an *alphabetic* writing system, can match these requirements, because they can use their unimpaired visuo-spatial and semantic pathway, while phonology plays a minor role, which we also found in the current study.

There was one remarkable, significant difference in the scanning behavior between the groups: Children of group C more often showed a linear analytic decoding behavior from left to right – using a proven strategy during scanning alphabetic words. Children of group DD more often located their first fixation the distinctive central location, which indicates a **visual-holistic** approach. Several studies have presented strong evidence that Chinese words are processed holistically^5^.

It has been reported recently that the manner of learning a logographic script influences the reading and writing performance^60^. As teaching in our study drew special attention to visual features of the characters, the visual-holistic approach might have been stimulated especially in the children with DD. From an educational and psychological perspective, our aim was to open new chances for children with DD by learning a new language in a different writing system without the problems they had in their alphabetic writing system.

The strengths of the study are that we recorded EM during naming tasks in two different writing systems in the same cohort of children and that we examined children with and without DD, who were educated in a regular alphabetic writing system. We examined their EM patterns based on their EM scan paths on the stimulus, which provided new insight into their processing strategy in a logographic writing system.

We analyzed their naming performance during tasks that required different skills and assessed phonological and visual aspects of processing the different stimuli.

Weaknesses of the current study are the limited number of participants. This was mainly caused by using strict inclusion and exclusion criteria, which we did to be able to work with a homogeneous group of children with DD without comorbidities. Furthermore, the limited number of stimuli did not allow forming subgroups of sufficient size within one stimulus type for statistical calculations. We decided on this number of stimuli, because we wanted to keep the durations of examinations short enough to maintain concentration and prevent fatigue during the experiments. This led to a rather descriptive approach to the analysis of the individual stimuli, which nevertheless allowed us to detect several frequently occurring performance patterns. Future studies with higher numbers of participants and stimuli would be desirable.

We are also aware of the possibility of results being impacted by participants’ familiarity with characters, which were introduced at different points in instruction. As a result, the children had different numbers of exposures to each character/opportunities to commit them to memory before testing.

## Conclusions

This study confirmed the high impact of the phonological deficit during alphabetic naming and showed the unimpaired visuo-spatial processing using the direct semantic pathway during naming pictures and Chinese characters, where, compared with alphabetic reading, phonological skills play a minor role. The majority of the children with DD located their first fixation in the center of the Chinese characters, indicating their holistic processing, whereas those without DD more often scanned from left to right using their linear- analytic mode as a proven strategy during alphabetic reading.

EM variables showed differences between the groups only for few individual Chinese characters, but percentage of correct answers was significantly lower in group DD, especially for naming Chinese characters in Chinese. Visual complexity had a moderate influence, differently in the groups: Higher visual complexity was associated with more fixations in group DD, and with longer latencies in group C. However, the higher error rate in group DD was neither correlated with visual complexity nor with measures of phonological deficit, but was associated with conspicuous features of individual characters. This indicates that other factors, besides phonology, have an impact, such as working memory, memorization, motivation and self-confidence. A longer learning period might have helped to overcome these obstacles.

The mainly unimpaired naming of the Chinese characters might open a chance for children with DD in an alphabetic writing system to learn a logographic script successfully. Future studies with a larger cohort of participants, more stimuli and a longer learning period could clarify these aspects.

## Electronic supplementary material

Below is the link to the electronic supplementary material.

Supplementary Material 1

Supplementary Material 2

Supplementary Material 3

## Data Availability

The datasets and raw data which were collected and analyzed during this study can be shared and are available from the corresponding author upon reasonable request. For privacy and data protection reasons, it is not possible to make this data publicly available.
