# Publisher Correction: Eye movements of children with and without developmental dyslexia in an alphabetic script during alphabetic and logographic tasks

**DOI:** 10.1038/s41598-025-88031-2

**Published:** 2025-03-28

**Authors:** Susanne Trauzettel-Klosinski, Theda Faisst, Vera Schick, Giulia Righetti, Christoph Braun, Angelika Cordey-Henke, Ching-Chu Sun, Stephan Kuester-Gruber

**Affiliations:** 1 Vision Rehabilitation Research Unit, Center for Ophthalmology, University of Tuebingen, Tuebingen, Germany; 2 Erich-Paulun-Institute, China Center Tuebingen, University of Tuebingen, Tuebingen, Germany; 3 Center for Ophthalmology, University Eye Hospital, University of Tuebingen, Tuebingen, Germany; 4 MEG-Center, University of Tuebingen, Tuebingen, Germany; 5 Hertie Institute for Clinical Brain Research, University of Tuebingen, Tuebingen, Germany; 6 DiPSCO, Department of Psychology and Cognitive Science, University of Trento, Rovereto, Italy; 7 Center for Mind/Brain Sciences, University of Trento, Rovereto, Italy; 8 Department of Quantitative Linguistics, University of Tuebingen, Tuebingen, Germany; 9 Department of Cognitive Science and Artificial Intelligence, Tilburg University, Tilburg, The Netherlands

Correction to: *Scientific Reports* 10.1038/s41598-024-78894-2, published online 20 November 2024

The original version of this Article contained errors in the ordering of References. References 17, 18 and 19 were incorrectly numbered as 51, 52 and 54.

The references have been reordered and cited accordingly.

The original Article has been corrected.

