# Supplementary material for: Eye movements of children with and without developmental dyslexia in an alphabetic script during alphabetic and logographic tasks
Source: Sci Rep. 2024 Nov 20;14:28796. doi: 10.1038/s41598-024-78894-2 (PMC11579334; doi:10.1038/s41598-024-78894-2)
Supplement: Supplementary file 1 — Supplementary Material 1 [file 41598_2024_78894_MOESM1_ESM.docx]

| ID | group | age [years] | sex | school  grade | reading speed [wpm] | reading errors | VA |
| --- | --- | --- | --- | --- | --- | --- | --- |
|  |  |  |  |  |  |  |  |
| 1 | DD | 10.4 | g | 5 | 73.3 | 12 | 1.40 |
| 2 | DD | 9.7 | b | 4 | 50.0 | 8 | 1.40 |
| 3 | DD | 11.3 | b | 5 | 54.0 | 8 | 1.40 |
| 4 | DD | 10.8 | b | 5 | 78.0 | 1 | 1.40 |
| 5 | DD | 10.1 | b | 4 | 71.0 | 9 | 1.25 |
| 6 | DD | 12.6 | b | 6 | 61.0 | 4 | 1.40 |
| 7 | DD | 10.1 | g | 4 | 39.0 | 2 | 1.40 |
| 8 | DD | 11.0 | b | 5 | 118.5 | 2 | 1.40 |
| 9 | DD | 10.9 | b | 5 | 21.4 | 26 | 1.40 |
| 10 | DD | 9.4 | g | 4 | 45.8 | 2 | 1.40 |
| 11 | DD | 11.2 | b | 5 | 56.0 | 0 | 1.25 |
| 12 | DD | 11.9 | b | 5 | 25.0 | 20 | 1.40 |
| 13 | DD | 11.5 | b | 5 | 107.0 | 8 | 1.40 |
| 14 | DD | 9.6 | b | 4 | 57.0 | 2 | 1.25 |
| 15 | DD | 10.1 | b | 4 | 79.0 | 4 | 1.40 |
| 16 | DD | 9.7 | b | 4 | 58.0 | 3 | 1.40 |
| 17 | DD | 10.1 | g | 4 | 44.0 | 3 | 1.25 |
| 18 | DD | 9.3 | b | 4 | 90.7 | 4 | 1.40 |
| 19 | C | 9.2 | b | 4 | 111.0 | 0 | 1.40 |
| 20 | C | 10.6 | b | 5 | 136.0 | 0 | 1.40 |
| 21 | C | 9.0 | g | 4 | 63.5 | 0 | 1.25 |
| 22 | C | 11.5 | g | 5 | 141.0 | 0 | 1.40 |
| 23 | C | 10.2 | g | 5 | 123.8 | 0 | 1.40 |
| 24 | C | 10.9 | b | 5 | 149.0 | 0 | 1.40 |
| 25 | C | 10.2 | b | 4 | 106.0 | 0 | 1.40 |
| 26 | C | 10.2 | b | 4 | 116.0 | 0 | 1.40 |
| 27 | C | 11.0 | g | 5 | 105.0 | 0 | 1.40 |
| 28 | C | 11.0 | g | 5 | 132.6 | 0 | 1.40 |
| 29 | C | 9.4 | g | 4 | 119.0 | 0 | 1.00 |
| 30 | C | 9.6 | b | 4 | 110.0 | 0 | 1.40 |
| 31 | C | 9.9 | b | 4 | 136.0 | 0 | 1.40 |
| 32 | C | 9.9 | g | 4 | 138.0 | 0 | 1.40 |
| 33 | C | 11.6 | g | 5 | 139.0 | 0 | 1.40 |
| 34 | C | 10.4 | b | 4 | 141.0 | 0 | 1.40 |
| 35 | C | 9.6 | g | 4 | 111.3 | 0 | 1.40 |
| 36 | C | 10.1 | b | 4 | 132.0 | 0 | 1.00 |
| 37 | C | 10.7 | g | 5 | 150.0 | 0 | 1.00 |
| 38 | C | 11.1 | g | 5 | 118.0 | 0 | 1.25 |
| 39 | C | 10.8 | g | 5 | 163.0 | 0 | 1.40 |
| 40 | C | 9.4 | b | 4 | 123.8 | 0 | 1.40 |

**Legend Table A1. Clinical data of all participants at baseline (modified after Kuester-Gruber, et al. 2023).** *DD: children with dyslexia (group DD), C: control (group C), g: girl, b: boy, reading speed and errors were s assessed by Zürcher reading test (ZLT II) in wpm (words per minute), VA (visual acuity, decimal)*
