# Supplementary material for: Eye movements of children with and without developmental dyslexia in an alphabetic script during alphabetic and logographic tasks
Source: Sci Rep. 2024 Nov 20;14:28796. doi: 10.1038/s41598-024-78894-2 (PMC11579334; doi:10.1038/s41598-024-78894-2)
Supplement: Supplementary file 2 — Supplementary Material 2 [file 41598_2024_78894_MOESM2_ESM.docx]

| **character** | **char. number** | **number of**  **strokes** | **number of**  **pixels** | **complexity** | **meaning in German (English)** |
| --- | --- | --- | --- | --- | --- |
| 人 | G1 | 2 | 1725 | + | Mensch, Person (human being, person) |
| 小 | C8 | 3 | 1961 | + | klein (small) |
| 大 | C1 | 3 | 2364 | + | groß (big) |
| 山 | C7 | 3 | 2511 | + | Berg (mountain) |
| 木 | G2 | 4 | 2674 | + | Holz, Baum (wood, tree) |
| 王 | G7 | 4 | 2767 | + | König (king) |
| 天 | G8 | 4 | 2842 | + | Himmel (sky) |
| 石 | C2 | 5 | 2981 | + | Stein (stone) |
| 龙 | G3 | 5 | 3256 | ++ | Drache (dragon) |
| 他 | C10 | 5 | 3577 | ++ | er (he) |
| 她 | G4 | 6 | 3876 | ++ | sie (she) |
| 鸡 | C9 | 7 | 3604 | ++ | Hahn / Huhn (rooster / chicken) |
| 我 | C3 | 7 | 3669 | ++ | ich (I) |
| 雨 | G9 | 8 | 3433 | ++ | Regen (rain) |
| 虎 | C4 | 8 | 3675 | ++ | Tiger (tiger) |
| 蛇 | G10 | 11 | 3733 | ++ | Schlange (snake) |
| 猫 | G11 / C5 | 11 | 4344 | +++ | Katze (cat) |
| 猪 | G12 / C6 | 11 | 4382 | +++ | Schwein (pig) |
| 猴 | C11 | 12 | 4403 | +++ | Affe (monkey) |
| 属 | G5 | 12 | 4563 | +++ | gehören zu [einem Tierkreiszeichen] (to belong to [a zodiac sign]) |
| 鼠 | G6 / C12 | 13 | 4132 | +++ | Ratte (rat) |

**Table A2.** **Chinese characters used in the Chinese and German naming tasks during eye tracking**, **here** arranged for all characters in the order of visual complexity. C= Chinese naming, G= German naming. The **numbers** of the individual characters are in a different order, because during eye tracking 4 separate blocks of characters were presented, each of them with increasing visual complexity: IR-ET: German naming: G1-G6, Chinese naming C1-C6, SLO: G7-G12, C7-C12.
