# Supplementary material for: Eye movements of children with and without developmental dyslexia in an alphabetic script during alphabetic and logographic tasks
Source: Sci Rep. 2024 Nov 20;14:28796. doi: 10.1038/s41598-024-78894-2 (PMC11579334; doi:10.1038/s41598-024-78894-2)
Supplement: Supplementary file 3 — Supplementary Material 3 [file 41598_2024_78894_MOESM3_ESM.docx]

| **character** | **pinyin (pronunciation)** | **meaning in German (English)** |
| --- | --- | --- |
| 1. 山 | shān | Berg (mountain) |
| 2. 人 | rén | Mensch, Person (human being, person) |
| 3. 水 | shuǐ | Wasser (water) |
| 4. 木 | mù | Holz, Baum (wood, tree) |
| 5. 天 | tiān | Himmel (sky) |
| 6. 大 | dà | groß (big) |
| 7. 他 | tā | er (he) |
| 8. 她 | tā | sie (she) |
| 9. 我 | wǒ | ich (I) |
| 10. 你 | nǐ | du (you) |
| 11. 是 | shì | sein (copula; "to be") |
| 12. 不 | bù | nicht / nein (not /no) |
| 13. 这 | zhè | dieser/diese/dieses (this/this/these) |
| 14. 那 | nà | jener/jene/jenes (that/those/those) |
| 15. 羊 | yáng | Schaf / Ziege (sheep / goat) |
| 16. 鸡 | jī | Hahn / Huhn (rooster / chicken) |
| 17. 猪 | zhū | Schwein (pig) |
| 18. 狗 | gǒu | Hund (dog) |
| 19. 猴 | hóu | Affe (monkey) |
| 20. 马 | mǎ | Pferd (horse) |
| 21. 蛇 | shé | Schlange (snake) |
| 22. 龙 | lóng | Drache (dragon) |
| 23. 兔 | tù | Hase (rabbit) |
| 24. 虎 | hǔ | Tiger (tiger) |
| 25. 喜 | xǐ | mögen (to like) |
| 26. 欢 | hūan | mögen (to like) |
| 27. 牛 | niú | Ochse / Büffel / Rind (Ox / buffalo / cattle) |
| 28. 鼠 | shǔ | Ratte (rat) |
| 29. 猫 | māo | Katze (cat) |
| 30. 小 | xiǎo | klein (small) |
| 31. 属 | shǔ | gehören zu [einem Tierkreiszeichen] (to belong to [a zodiac sign]) |
| 32. 王 | wáng | König (king) |
| 33. 雨 | yǔ | Regen (rain) |
| 34. 石 | shí | Stein (stone) |
| 35. 臣 | chén | Minister (minister) |
| 36. 竹 | zhú | Bambus (bamboo) |
| 37. 川 | chūan | Fluss (river) |

**Table A3.** **All Chinese characters taught during the lessons are shown in the order, in which they were presented in class.**
